# Uncertainty in perception and the Hierarchical Gaussian Filter

**DOI:** 10.3389/fnhum.2014.00825

**Published:** 2014-11-19

**Authors:** Christoph D. Mathys, Ekaterina I. Lomakina, Jean Daunizeau, Sandra Iglesias, Kay H. Brodersen, Karl J. Friston, Klaas E. Stephan

**Affiliations:** ^1^Wellcome Trust Centre for Neuroimaging, Institute of Neurology, University College LondonLondon, UK; ^2^Max Planck UCL Centre for Computational Psychiatry and Ageing ResearchLondon, UK; ^3^Translational Neuromodeling Unit, Institute for Biomedical Engineering, University of Zurich and ETH ZurichZurich, Switzerland; ^4^Laboratory for Social and Neural Systems Research (SNS Lab), Department of Economics, University of ZurichZurich, Switzerland; ^5^Department of Computer Science, ETH ZurichZurich, Switzerland; ^6^Institut du Cerveau et de la Moelle Épinière, Hôpital Pitié SalpêtrièreParis, France

**Keywords:** uncertainty, volatility, Bayesian inference, hierarchical modeling, filtering, free energy, learning, decision-making

## Abstract

In its full sense, perception rests on an agent's model of how its sensory input comes about and the inferences it draws based on this model. These inferences are necessarily uncertain. Here, we illustrate how the Hierarchical Gaussian Filter (HGF) offers a principled and generic way to deal with the several forms that uncertainty in perception takes. The HGF is a recent derivation of one-step update equations from Bayesian principles that rests on a hierarchical generative model of the environment and its (in)stability. It is computationally highly efficient, allows for online estimates of hidden states, and has found numerous applications to experimental data from human subjects. In this paper, we generalize previous descriptions of the HGF and its account of perceptual uncertainty. First, we explicitly formulate the extension of the HGF's hierarchy to any number of levels; second, we discuss how various forms of uncertainty are accommodated by the minimization of variational free energy as encoded in the update equations; third, we combine the HGF with decision models and demonstrate the inversion of this combination; finally, we report a simulation study that compared four optimization methods for inverting the HGF/decision model combination at different noise levels. These four methods (Nelder–Mead simplex algorithm, Gaussian process-based global optimization, variational Bayes and Markov chain Monte Carlo sampling) all performed well even under considerable noise, with variational Bayes offering the best combination of efficiency and informativeness of inference. Our results demonstrate that the HGF provides a principled, flexible, and efficient—but at the same time intuitive—framework for the resolution of perceptual uncertainty in behaving agents.

## Introduction

Perception has long been proposed to take place in the context of prediction (Helmholtz, 1860). This entails that agents have a model of the environment which generates their sensory input. Probability theory formally prescribes how agents should learn about their environment from sensory information, given a model. This rests on sequential updating of beliefs according to Bayes' theorem, where beliefs represent inferences about hidden states of the environment in the form of posterior probability distributions. It is this process that we refer to as perception. Beliefs about hidden states are inherently uncertain. This uncertainty has two sources. First, even when states are constant, the amount of sensory information will in general be too little to infer them exactly. This has been referred to as *informational uncertainty* or *estimation uncertainty* (Payzan-LeNestour and Bossaerts, 2011). The second source of uncertainty is the possibility that states change with time, i.e., *environmental uncertainty*.

Various models have suggested how an agent may deal with an environment fraught with both kinds of uncertainty (e.g., Yu and Dayan, 2003, 2005; Nassar et al., 2010; Payzan-LeNestour and Bossaerts, 2011; Wilson et al., 2013). Here, we discuss an alternative approach that derives closed form update equations for the hidden states, and crucially, for the uncertainty about them, by variational inversion of a generic hierarchical generative model that reflects the time-varying structure of the environment in its higher levels. This derivation has the advantage that the resulting updates optimize a clearly defined objective function, namely variational free energy. Since this quantity is an approximation to surprise (i.e., to the negative log-probability of sensory input), the updates are optimal in the sense that they minimize surprise, given an agent's individual model. Furthermore, the updates explicitly reflect informational and environmental uncertainty.

Our approach makes use of a framework which assumes that agents have an internal generative model of their sensory input. This model is generative in the sense that it describes how sensory inputs are generated by the external world. It does this by assigning a probability (the likelihood) to each sensory input given states (which vary with time) and parameters (which are constant in time) and by completing this with a prior probability distribution for states and parameters. While the purpose of the model is to predict input emanating from the external world, it is internal in the sense that it reflects the agent's *beliefs* about how sensory inputs are generated by the external world.

While Bayesian belief updating is optimal from the perspective of probability theory, it requires computing complicated integrals which are not tractable analytically and difficult to evaluate in real time. Although some attempts to design a Bayesian model of how biological agents learn in a changing environment were remarkably successful in explaining empirical behavior (Behrens et al., 2007, 2008), they were restricted by the computational burden imposed by these models and the assumption that the learning process was identical across subjects. Recently, however, theoretical advances have enabled computationally efficient approximations to exact Bayesian inference during learning (e.g., Friston, 2009; Daunizeau et al., 2010a,b) and have furnished a basis for biologically plausible mechanisms that might underlie belief updating in the brain. These approaches rest on variational Bayesian techniques which optimize a free-energy bound on the surprise about sensory inputs, given a model of the environment, and represent a special case of the general “Bayesian brain” hypothesis (Dayan et al., 1995; Knill and Pouget, 2004; Körding and Wolpert, 2006; Friston, 2009; Doya et al., 2011). This hypothesis has been highly influential in recent years, shaping concepts of brain function and inspiring the design of many specific computational models (see Friston and Dolan, 2010, for review). However, for practical applications to empirical data, a general purpose modeling framework has been lacking that would allow for straightforward “off the shelf” implementations of models explaining trial-wise empirical data (e.g., behavioral responses, eye movements, evoked response amplitude in EEG etc.) from the Bayesian brain perspective. This is in contrast to reinforcement learning (RL) models which, due to their simplicity and computational efficiency, have found widespread application in experimental neuroscience, for example, in the analysis of functional magnetic resonance imaging (fMRI) and behavioral data (for reviews, see Daw and Doya, 2006; O'Doherty et al., 2007).

To fill this gap and provide a generic, robust and flexible framework for analysis of trial-wise data from the Bayesian brain perspective, we recently introduced the Hierarchical Gaussian Filter (HGF), a hierarchical Bayesian model Mathys et al. (2011) in which states evolve as coupled Gaussian random walks, such that each state determines the step size of the evolution of the next lower state (for examples of applications, cf. Iglesias et al., 2013; Joffily and Coricelli, 2013; Vossel et al., 2013). Based on a mean field approximation to the full Bayesian solution, we derived analytic update equations whose form resembles RL updates, with dynamic learning rates and precision-weighted prediction errors. These highly efficient update equations made our approach well suited for filtering purposes, i.e., predicting the value of (and, crucially, the uncertainty about) a hidden and moving quantity based on all information acquired up to a certain point. Our original formulation (Mathys et al., 2011) only contained three levels; here, we extend the HGF explicitly to any number of levels and show that the update equations maintain the same form across all levels because they are derived on the basis of the same coupling. Furthermore, the derivation of the variational energies involved in the inversion is given in much more detail than in Mathys et al. (2011). It is important to note that “perceptual uncertainty” has a broader meaning here than in Mathys et al. (2011), where it was used more narrowly for that part of the informational uncertainty that relates to sensory input.

Furthermore, in this paper we describe how the HGF is applied in the “observing the observer” framework developed in (Daunizeau et al., 2010a,b). This framework is based on a clear separation of two model components: First, the agent's perception of (inference about) its environment, i.e., the posterior estimates provided by the agent's model of how its sensory input is generated. Second, the agent's observed actions (i.e., decisions or responses) which are (probabilistic) consequences of the agent's beliefs about its environment. We call the first model *perceptual*, while the second is the *decision* or *response* model.

The “observing the observer” framework is meta-Bayesian in that it enables Bayesian inference (by an observer or experimenter) on Bayesian inference (by a subject). It requires four elements: (1) a generative model of sensory inputs (i.e., a perceptual model), (2) a computationally efficient and robust method for model inversion, (3) a loss function for actions depending on the inferred state, and (4) a decision model. A specific suggestion for the first two elements is contained in Mathys et al. (2011). In what follows, we extend and generalize this description, discussing specifically the nature of the coupling between levels, choice of coordinates at higher levels, and how to deal with sensory inputs that arrive at irregular time intervals.

In the following section of this paper, we set out our theoretical framework. We first define the HGF model formally. We then proceed with its variational inversion, which gives us closed one-step update equations. Next, we show how the HGF can serve as a perceptual model for any decision model that provides a mapping from the HGF's representations of the environment to the probability of an observed decision. We then derive an objective function whose optimization leads to maximum-a-posterior (MAP) estimates for the parameters of the HGF and the decision model, followed by a short discussion of how the choice of decision model affects which HGF parameters can be estimated.

In the next section, we turn to examples and simulations. We first deal with categorical outcomes and sensory uncertainty. To complement this, we introduce a decision model for binary choices and use it to give an example of model inversion and comparison based on two different but closely related decision models. We do this by estimating model parameters from empirical data in a single subject, juxtaposing the two different response models (which do and do not take into account the uncertainty of beliefs) and the ensuing differences in inferred state trajectories. Next, we introduce a decision model for a one-armed bandit. As in the preceding examples of decision models, we base the decision rule on the agent's expected loss under a given loss function. In the last part of this section, we report the results of a simulation study which demonstrates the feasibility of accurate model inversion, i.e., inferring known HGF parameter values from observed decisions. This test of model inversion was performed under different levels of noise and using four different optimization methods (Markov chain Monte Carlo, the Nelder–Mead simplex algorithm, Gaussian process-based global optimization, and variational Bayes.

Together, the theoretical derivations and simulation results provided in this paper generalize the framework of the HGF and demonstrate its utility for estimating individual approximations to Bayes-optimality from observed decision-making under uncertainty.

## Theoretical framework

### The hierarchical gaussian filter (HGF)

The goal of the model introduced in Mathys et al. (2011) is simple and general: to describe how an agent learns about a continuous uncertain quantity (i.e., random variable) *x* that moves. One generic way of describing this motion is a Gaussian random walk:

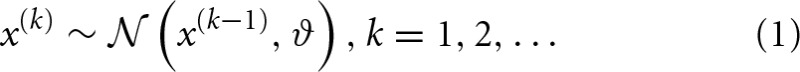



where *k* is a time index, and *x*^(*k* − 1)^ and ϑ are the mean and variance (not standard deviation) of a Gaussian distribution, respectively. In this formulation, the volatility in *x* is governed by the positive constant ϑ (in this paper, we define volatility as the variance of a time series per unit of time); however, there is in principle no reason to assume that volatility is constant. To allow for changes in volatility, we replace ϑ by a positive function *f* of a second random variable, *x*_2_, while *x* becomes *x*_1_:

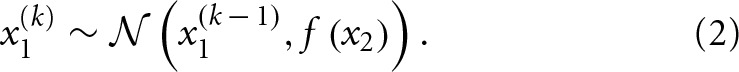



**Figure 1:**
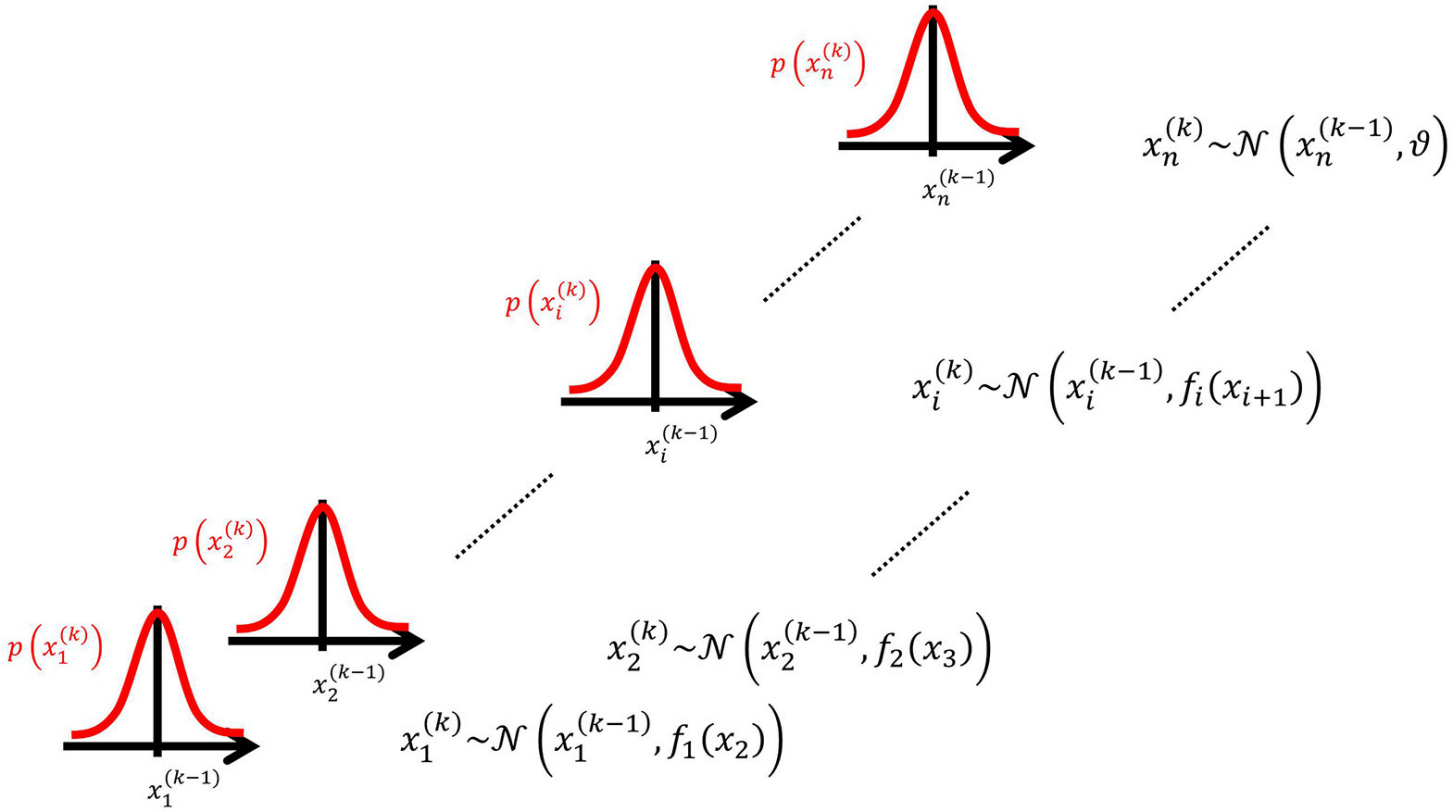
**Overview of the Hierarchical Gaussian Filter (HGF)**. The model represents a hierarchy of coupled Gaussian random walks. The levels of the hierarchy relate to each other by determining the step size (volatility or variance) of a random walk. The topmost step size is a constant parameter ϑ.

We may now further assume that *x*_2_ performs a Gaussian random walk of its own, with a constant variance ϑ, so that the model for *x*_2_ is the same as the one for *x* in Equation 1. This adding of levels of Gaussian random walks coupled by their variances can now continue up to any number *n* of levels in the hierarchy, as illustrated in Figure 1. At each level *i*, the coupling to the next highest level *i* + 1 is given by a positive function *f_i_*(*x*_*i* + 1_) which represents the variance or step size of the random walk:

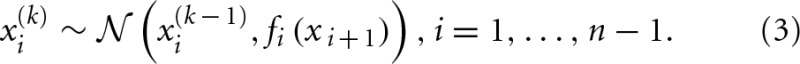



At the top level, instead of *f_n_*, we have ϑ:

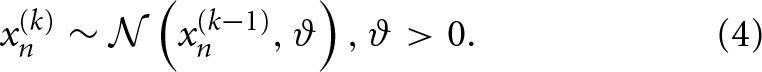



To complete our model, we still need to define the *f_i_* in Equation 3. A flexible and straightforward approach to this is to allow any positive analytic *f_i_*, but to expand it in powers to first order to give it a simple functional form. However, since *f_i_* has to be positive, we cannot approximate it by expanding it directly. Instead, dropping indices for clarity, we expand its logarithm (for details, see Appendix A), which motivates our definition of coupling between levels:

(5) $$f_{i}\left(x_{i+1}\right)\overset{\text{def}}{=}\exp\left(\kappa_{i}x_{i+1}+\omega_{i}\right)$$

As we will see below, this form of coupling has the additional advantage of enabling the derivation of simple one-step update equations under a mean-field approximation.

By further assuming that inputs (observations) *u*^(*k*)^ are generated by means of a Gaussian emission distribution of the form.

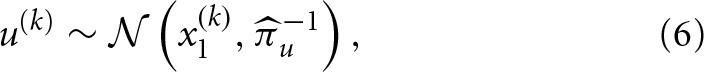



where $\hat{\pi }$_*u*_ denotes the precision of the emission distribution, the model defined by Equations 3 and 5 can be used for online prediction of *x*^(*k*)^_1_, i.e., *filtering*. Considering that the model consists of a hierarchy of Gaussian random walks, this motivates why we refer to it as the *HGF*.

To illustrate this, we might imagine a time series of financial data. *u*^(*k*)^ could be the observed returns of a particular security. *x*^(*k*)^_1_ then is the underlying quantity (the true return after observation noise $\hat{\pi }$^−1^_*u*_ has been filtered out). ω_*i*_ is the tonic (i.e., time-invariant) part of the (log-)volatility of *x*_1_, while κ*x*_2_ is the phasic (i.e., time-varying) part. Accounting for the scaling by κ, *x*_2_ is now the scaled phasic log-volatility of *x*_1_. This pattern then repeats until the top of the hierarchy is reached. One of the advantages of the HGF is that volatility is captured hierarchically: not only returns are volatile, but also their volatility, and the volatility of the volatility, etc.

### Approximate inversion for sensory input at irregular intervals

Sensing takes place at the bottom of the hierarchy: *u* is the agent's sensory input. To allow for input that comes at irregular intervals, we can multiply the variance of the random walks at all levels by the time *t*^(*k*)^ that elapses between the arrival of inputs *u*^(*k* − 1)^ and *u*^(*k*)^:

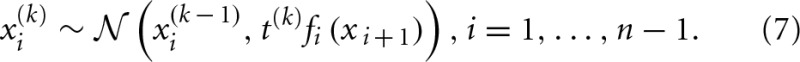



This proportionality of the variance to time reflects the fact that the mean squared distance of a quantity performing a Gaussian random walk from its origin is proportional to time (cf. the connection between Gaussian random walks, Brownian motion, and the heat equation; Evans, 2010). For inputs at regular intervals, we may simply set *t*^(*k*)^ = 1 for all *k*, effectively removing *t*^(*k*)^ from the model.

We can now derive update equations using the variational inversion method introduced in Mathys et al. (2011). This approximate inversion assumes Gaussian posteriors at all levels with means μ_*i*_ and precisions (inverse variances) π_*i*_:

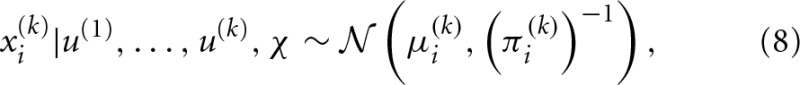



where χ $\underset{=}{\text{def}}$ {κ, ω, …, κ_*n*−1_, ω_*n*−1_, ϑ}. This is an approximation because the true posterior distribution *p*(*x*^(*k*)^_*i*_|*u*^(1)^, …, *u*^(*k*)^, χ) will deviate somewhat from a Gaussian shape. A discussion of the variational nature and the implications of this approximation can be found in Mathys et al. (2011). The sufficient statistics μ_*i*_ and π_*i*_ are the quantities that are updated after each new input *u* according to the following equations (cf. the Discussion, where we give a natural interpretation of them in terms of learning rate and prediction error):

(9) $$\mu_{i}^{\left(k\right)}={\hat{\mu }}_{i}^{\left(k\right)}\text{+}\frac{1}{2}\kappa_{i-1}\;v_{i-1}^{\left(k\right)}\frac{{\hat{\pi }}_{i-1}^{\left(k\right)}}{\pi_{i}^{\left(k\right)}}\delta_{i-1}^{\left(k\right)}$$

(10) $$\begin{array}{l} \pi_{i}^{\left(k\right)}={\hat{\pi }}_{i}^{\left(k\right)}+\frac{1}{2}{\left(\kappa_{i-1}v_{i-1}^{\left(k\right)}{\hat{\pi }}_{i-1}^{\left(k\right)}\right)}^{2}\\ \;\left(1+\left(1-\frac{1}{v_{i-1}^{\left(k\right)}\pi_{i-1}^{\left(k-1\right)}}\right)\delta_{i-1}^{\left(k\right)}\right) \end{array}$$

with

(11) $$v_{i}^{\left(k\right)}\overset{\text{def}}{=}\{\begin{array}{c} t^{\left(k\right)}\exp\left(\kappa_{i}\mu_{i+1}^{\left(k-1\right)}+\omega_{i}\right),i=1,\ldots ,n-1\\ t^{\left(k\right)}\vartheta ,\;i=n \end{array}$$

(12) $${\hat{\mu }}_{i}^{\left(k\right)}\overset{\text{def}}{=}\mu_{i}^{\left(k-1\right)}$$

(13) $${\hat{\pi }}_{i}^{\left(k\right)}\overset{\text{def}}{=}\frac{1}{\sigma_{i}^{\left(k-1\right)}+v_{i}^{\left(k\right)}}$$

(14) $$\delta_{i}^{\left(k\right)}\overset{\text{def}}{=}\frac{\sigma_{i}^{\left(k\right)}+{\left(\mu_{i}^{\left(k\right)}-{\hat{\mu }}_{i}^{\left(k\right)}\right)}^{2}}{\sigma_{i}^{\left(k-1\right)}+v_{i}^{\left(k\right)}}-1$$

Variances (i.e., inverse precisions) are denoted by σ^(*k*)^_i_ = 1/π^(*k*)^_*i*_. Note that irregular intervals between inputs are fully accounted for by the factor *t*^(*k*)^ in Equation 11. While the updates of Equations 9 and 10 apply to all but the first level, they are different at the first level:

(15) $$\mu_{1}^{\left(k\right)}={\hat{\mu }}_{1}^{\left(k\right)}+\frac{{\hat{\pi }}_{u}}{\pi_{1}^{\left(k\right)}}\delta_{u}^{\left(k\right)}$$

(16) $$\pi_{1}^{\left(k\right)}={\hat{\pi }}_{1}^{\left(k\right)}+{\hat{\pi }}_{u}$$

with

(17) $$\delta_{u}^{\left(k\right)}\overset{\text{def}}{=}u^{\left(k\right)}-{\hat{\mu }}_{1}^{\left(k\right)}$$

where $\hat{\mu }$^(*k*^)_1_ and $\hat{\pi }$^(*k*)^_1_ are defined by Equations 12 and 13. The different form of the updates at the first level arises because, at the first level, the direction of inference is from *u* to *x*_1_, which appears in the *mean* of the Gaussian in Equation 6, while at all higher levels, the direction of inference is from *x*_i_ to *x*_*i*_ + 1, which appears in the *variance* of the Gaussian in Equation 3. This results in the updates being driven by different kinds of prediction errors: value prediction errors (VAPEs) at the first level, volatility (i.e., variance) prediction errors (VOPEs) at all higher levels. We elaborate this distinction in the Discussion below.

The details of this inversion are given in Appendix B. The notation chosen here emphasizes the role of precisions in the updating process more than that used in (Mathys et al., 2011); they are, however, equivalent.

### Maximum-a-posteriori (MAP) parameter estimation

Given the above update equations, initial representations $\lambda^{\left(0\right)}\overset{\text{def}}{=}\left\{\mu_{1}^{\left(0\right)},\pi_{1}^{\left(0\right)},\ldots ,\mu_{n}^{\left(0\right)},\pi_{n}^{\left(0\right)}\right\}$(i.e., the means μ_*i*_ and precisions π_*i*_ of the states *x_i_* at time *k* = 0), and priors on the perceptual parameters χ, we could invert the model (i.e., estimate the values of χ that lead to least aggregate surprise about sensory inputs) from sensory inputs alone; this would provide us with state trajectories and parameters which represent an ideal Bayesian agent, where “ideal” means experiencing the least surprise about sensory inputs. However, our goal is usually different; it is to estimate subject-specific parameters (which encode the individual's approximation to Bayes-optimality) from his/her observed behavior, as formalized in the “observing the observer” framework (Daunizeau et al., 2010a,b). To achieve this goal, we will now bring the HGF into this framework. This requires the introduction of a response model which links the agent's current estimates $\lambda^{\left(k\right)}\overset{\text{def}}{=}\left\{\mu_{1}^{\left(k\right)},\sigma_{1}^{\left(k\right)},\ldots ,\mu_{n}^{\left(k\right)},\sigma_{n}^{\left(k\right)}\right\}$ of states to expressed decisions *y*^(*k*)^ and which also contains subject-specific parameters $\zeta \;\overset{\text{def}}{=}\left\{\zeta_{1},\zeta_{2},\ldots \right\}$. For example, a useful response model (e.g., Iglesias et al., 2013), which we also use in our simulation study below, is the unit-square sigmoid, which maps the predictive probability *m^(k)^* that the next outcome will be 1 onto the probabilities *p*(*y*^(*k*)^ = 1) and *p*(*y*^(*k*)^ = 0) that the agent will choose response 1 or 0, respectively:

(18) $$p\left(y|m,\zeta \right)\text{​}=\text{​​}{\left(\frac{m^{\zeta }}{m^{\zeta }+\text{​}{\left(1-m\text{​}\right)}^{\zeta }}\text{​}\right)}^{y}\cdot \text{​}{\left(\frac{\text{​}{\left(1-m\text{​}\right)}^{\zeta }}{m^{\zeta }+\text{​}{\left(1-m\text{​}\right)}^{\zeta }}\text{​}\right)}^{1-y},$$

where, for clarity, we have omitted time indices on *y* and *m*. For this to serve as a response model for the inversion of the HGF, the predictive probability *m* = *m*(λ) has to be a function of the quantities λ the HGF keeps track of. This model is explained and discussed in detail below. **Figure 4** is a graphical representation of it. For our present purposes, the only important point is that it contains a parameter ζ that captures how deterministically *y* is associated with *m*. The higher ζ, the more likely the agent is to choose the option that is more likely according to its current belief. Since *m* is a deterministic function of λ, we can write *p*(*y* | *m*, ζ) = *p*(*y* | λ, ζ).

In general, the joint distribution for observations (i.e., decisions) and parameters of an HGF-based decision model takes the form

(19) $$\begin{array}{l} p\left(y,\chi ,\lambda^{\left(0\right)},\zeta |u\right)=p\left(\chi ,\lambda^{\left(0\right)},\zeta \right)\\ \;\prod_{k=1}^{K}p\left(y^{\left(k\right)}|\lambda^{\left(k\right)}\left(\chi ,\lambda^{\left(0\right)},u\right),\zeta \right) \end{array}$$

where $u\overset{\text{def}}{=}\left\{u^{\left(1\right)},\ldots ,u^{\left(K\right)}\right\}$ and $y\overset{\text{def}}{=}\left\{y^{\left(1\right)},\ldots ,y^{\left(K\right)}\right\}$ are the inputs and responses at time points *k* = 1 to *k* = *K*, respectively, and λ^(*k*)^(χ, λ^(0)^, *u*) are the sufficient statistics $\lambda^{\left(k\right)}\overset{\text{def}}{=}\left\{\mu_{1}^{\left(k\right)},\sigma_{1}^{\left(k\right)},\ldots ,\mu_{n}^{\left(k\right)},\sigma_{n}^{\left(k\right)}\right\}$ of the hidden states of the HGF at time *k*. The inputs *u* are given because the agent and its observer both know them and the agent uses them to invert its HGF model, resulting in the trajectories λ^(*k*)^. This makes it clear that the decision model is not a generative model of sensory input, while the HGF is. Strictly speaking, the decision model does not use the perceptual model directly. Instead, the decision model uses the perceptual model indirectly via its inversion, where input is given. It is also noteworthy that the sets of inputs *u* and observations *y* are finite here (*k* = 1,…, *K*), while the HGF is open-ended (cf. *k* = 1, 2, … in Equation 1).

The goal now is to find an expression for the maximum-a-posteriori (MAP) estimate for the parameters $\xi \;\overset{\text{def}}{\text{=}}\left\{\chi ,\lambda^{\left(0\right)},\zeta \right\}$. The MAP estimate ξ^*^ of ξ is defined as

(20) $$\xi^{*}\overset{\text{def}}{=}\underset{\xi }{\text{arg max}}p(\xi |y,u),$$

We unpack this in Appendix E to make it tractable:

(21) $$\begin{array}{l} \xi^{*}=\underset{\xi }{\text{arg max}}(\sum_{k}\ln p\left(y^{\left(k\right)}|\lambda^{\left(k\right)}\left(\chi ,\lambda^{\left(0\right)},u\right),\zeta \right)\\ \;+\ln p(\xi )) \end{array}$$

The objective function *Z*(ξ) that needs to be maximized is therefore the log-joint probability density of the parameters ξ and responses *y* given inputs *u*:

(22) $$\begin{array}{l} Z\left(\xi |u,y\right)\overset{\text{def}}{=}\ln p\left(\xi ,y|u\right)\\ \;=\sum_{k}\ln p\left(y^{\left(k\right)}|\lambda^{\left(k\right)}\left(\chi ,\lambda^{\left(0\right)},u\right),\zeta \right)+\ln p(\xi ) \end{array}$$

While the response model gives *p*(*y*^(*k*)^ | λ^(*k*)^, ζ), the perceptual model (i.e., the HGF) provides the representations λ^(*k*)^(χ, λ^(0)^, *u*) by way of its update equations. The last missing part in Equation 22 is the prior distribution *p*(ξ). This will be discussed below. There are many alternative optimization procedures to implement the maximization in Equation 21. We have compared four in the simulations discussed below.

Finally, one important point in relation to model inversion is model identifiability which we discuss in detail in Appendix F. In brief, when the posterior mean of the state at level *i* (i.e., μ_*i*_) is included in the response model and thus affects measured behavior, all quantities at that level μ^(0)^_*i*_, κ_*i*_, and ω_*i*_ can be estimated. If μ_*i*_ is not included in the response model, it is advisable to fix two of the three parameters μ^(0)^_*i*_, κ_*i*_, and ω_*i*_, reflecting a particular choice of origin and scale on *x_i_*. This avoids an overparameterized model.

### Priors and transformed parameter spaces

A crucial part of Bayesian inference is the specification of a prior distribution, in our case *p*(ξ). There is no principled reason why the priors on the different elements of ξ should not be independent; therefore, we may assume the following factorization:

(23) $$p(\xi )=p(\vartheta )\prod_{i}p(\kappa_{i})p(\omega_{i})p\left(\mu_{i}^{\left(0\right)}\right)p\left(\sigma_{i}^{\left(0\right)}\right)\prod_{j}p\left(\zeta_{j}\right).$$

*p*(ζ_*j*_) depends on the response model chosen and will have to be dealt with on a case-by-case basis (see below), but the remaining marginal priors are generic and will be discussed in what follows.

The most straightforward case are the priors on ω_*i*_. Since ω_*i*_ can take values on the whole real line, it can be estimated in its native space with a (possibly wide) Gaussian prior:

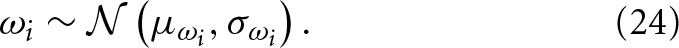



The same applies to μ^(0)^_*i*_:

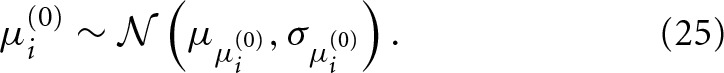



σ^(0)^_*i*_ has a natural lower bound at zero since it is a variance. We can avoid non-positive values by estimating σ^(0)^_*i*_ in log-space. That is, we use a log-Gaussian prior:

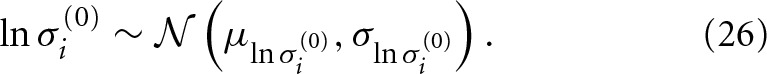



Just like σ^(0)^_*i*_, ϑ is a variance and has a lower bound at zero. In addition to the lower bound, it is desirable to have an upper bound on ϑ because, for a ϑ too large, the assumptions underlying the derivation of the update equations of the HGF no longer hold. Specifically, for large values of ϑ it is possible to get updates that push the precision π_*n*_ at the top level below zero, indicating that the agent knows “less than nothing” about *x*_*n*_. In less extreme cases, a large ϑ may allow μ_*n*_ to jump to very high levels, giving rise to improbable inference trajectories (cf. Equations 9 and 11). This is due to a violation of the assumption that the variational energies *I*(*x*_*i*_) are nearly quadratic (see Mathys et al., 2011, for details).

To avoid such violations, it is sensible to place an upper bound on ϑ in addition to the lower bound at zero. This can be achieved by estimating ϑ in “logit-space,” a logistic sigmoid transformation of native space with a variable upper bound *a* > 0:

(27) $$\begin{array}{l} {\text{logit}}_{a}(x)\overset{\text{def}}{=}\ln\left(\frac{x}{a-x}\right);\\ \;\Rightarrow x\;=\frac{a}{1+\exp\left(-{\text{logit}}_{a}(x)\right)} \end{array}$$

In that space, the prior on ϑ can then be taken as

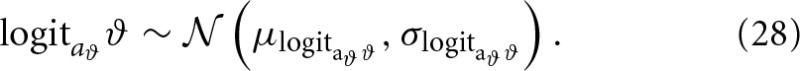



While κ_*i*_ can in principle take any real value, flipping the sign of κ_*i*_ is equivalent to flipping that of *x*_*i*+1_ (cf. Equation 5). It is therefore useful to adopt the convention that all κ_*i*_ > 0. This leads to the intuitive relation that a greater *x*_*i*+1_ means a greater variability in *x_i_*; in other words, this makes *f_i_* in Equation 3 a monotonically increasing function. A second useful constraint on κ_*i*_ is that it is bounded above, for the same reason as ϑ. Consequently, we evaluate κ_*i*_ in logit-space with the following priors:

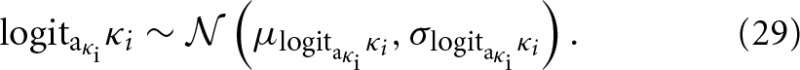



The exact specification of the above priors can vary, depending on the experimental context and instructions given to the subject (e.g., whether or not to expect a volatile environment). In most cases, a choice of κ_*i*_ and ϑ with upper bounds at or below 2 will be sensible. In cases where there is little movement in *x_n_* (the topmost *x*), a choice of ϑ closer to 0 than 1 will be appropriate. Notably, given that choosing a different prior amounts to having a different model, the choice between alternative priors can be evaluated using model comparison (cf. Stephan et al., 2009).

## Examples and simulations

### Categorical outcomes and sensory uncertainty

In the formulation above, *x*_1_ performs a Gaussian random walk on a continuous scale. However, states of an agent's environment that generate sensory input are often categorical, in the simplest case binary (e.g., present/absent). This fact can be accommodated in the perceptual model above by making the base level, *x*_1_, binary (we omit time indices *k* unless they are needed to avoid confusion):

(30) $$x_{1}\in \left\{0,1\right\}.$$

The second level, *x*_2_, of the model then describes the tendency of *x*_1_ toward state 1:

(31) $$p\left(x_{1}|x_{2}\right)=s{\left(x_{2}\right)}^{x_{1}}{\left(1-s(x_{2})\right)}^{1-x_{1}}\text{=Bernoulli}(x_{1};s(x_{2}))$$

where $s(x_{2})\overset{\text{def}}{=}\frac{1}{1+\exp(-x_{2})}$ is the logistic sigmoid function. Similarly, when *x*_1_ represents *d* > 2 outcomes, the probability of each can be represented by its own *x*_2_, performing (at most) *d* − 1 independent random walks. *p*(*x*_1_ = 0) simply is 1 − *p*(*x*_1_ = 1).

**Figure 2:**
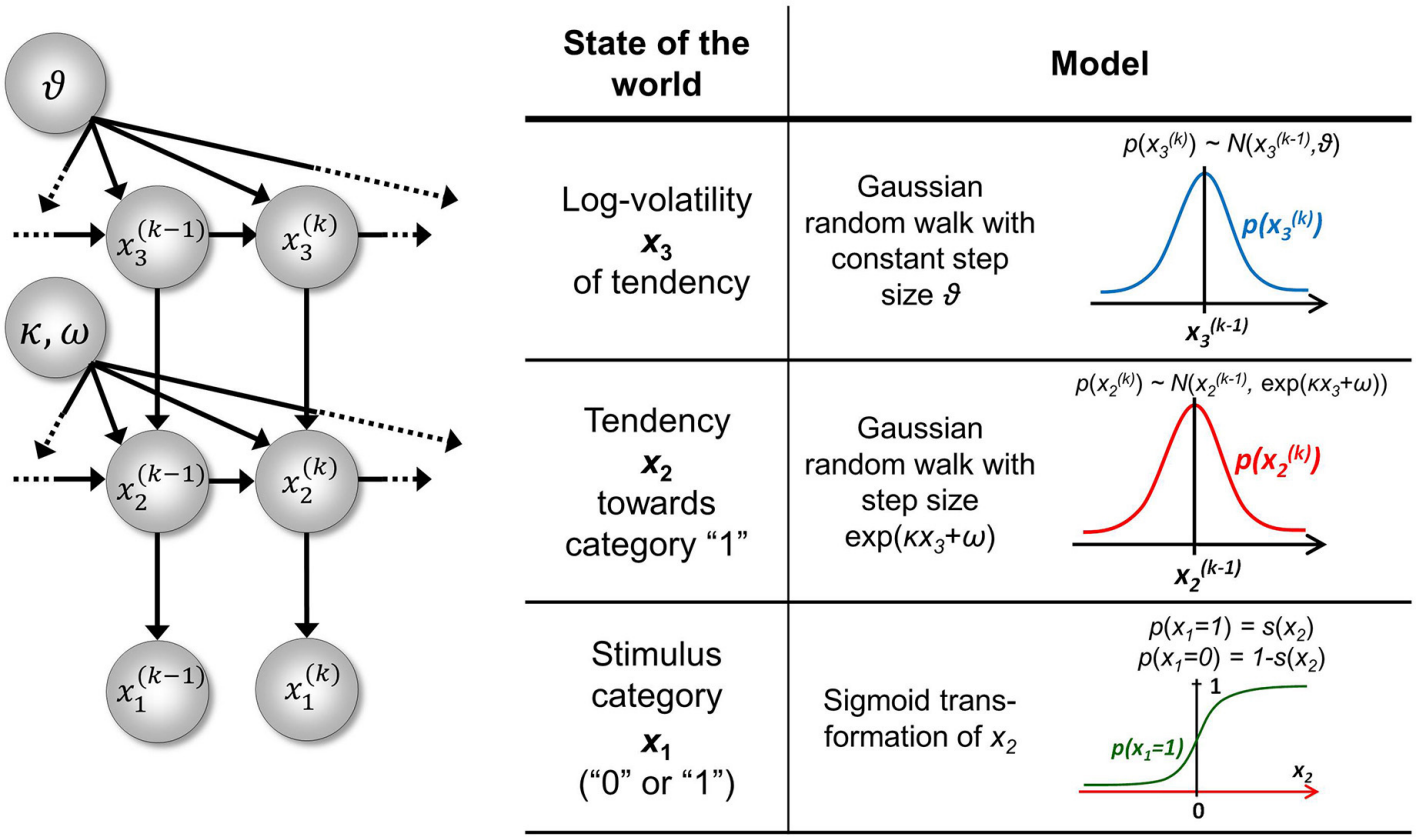
**The 3-level HGF for binary outcomes**. The lowest level, *x*_1_, is binary and corresponds, in the absence of sensory noise, to sensory input *u*. Left: schematic representation of the generative model as a Bayesian network. *x*^(k)^_1_, *x*^(*k*)^_2_, *x*^(*k*)^_3_ are hidden states of the environment at time point *k*. They generate *u*^(*k*)^, the input at time point *k*, and depend on their immediately preceding values *x*^(k − 1)^_2_, *x*^(*k* −1)^_3_ and on the on parameters κ, ω, ϑ. Right: model definition. This figure has been adapted from Figures 1, 2 in Mathys et al. (2011).

In the three-level HGF for binary outcomes (Mathys et al., 2011) the third level, *x*_3_ is at the top, with constant step variance ϑ. The only level with a coupling of the form of Equation 5 is therefore the second level; this allows us to write κ_2_ ≡ κ and ω_2_ ≡ ω. We can allow for sensory uncertainty by including an additional level at the bottom of the hierarchy that predicts sensory input *u* from the state *x*_1_. In the absence of sensory uncertainty, knowledge of the state *x*_1_ enables accurate prediction of input *u* and vice versa; we may then simply set *u* ≡ *x*_1_ and treat *x*_1_ as if it were directly observed. A graphical overview of this model is given in Figure 2.

For the particular case of the three-level HGF for binary outcomes, the general update equations in Equations 9–14 (with *t*^(*k*)^ = 1 for all *k*) take the following specific form (as previously derived in (Mathys et al., 2011), with additional detail in Appendix D):

(32) $$\mu_{2}^{\left(k\right)}=\mu_{2}^{\left(k-1\right)}+\frac{1}{\pi_{2}^{\left(k\right)}}\delta_{1}^{\left(k\right)}$$

(33) $$\pi_{2}^{\left(k\right)}={\hat{\pi }}_{2}^{\left(k\right)}+\frac{1}{{\hat{\pi }}_{1}^{\left(k\right)}}.$$

with

(34) $${\hat{\mu }}_{1}^{\left(k\right)}\overset{\text{def}}{=}s\left(\mu_{2}^{\left(k-1\right)}\right)$$

(35) $$\delta_{1}^{\left(k\right)}\overset{\text{def}}{=}\mu_{1}^{\left(k\right)}-{\hat{\mu }}_{1}^{\left(k\right)}$$

(36) $${\hat{\pi }}_{1}^{\left(k\right)}\overset{\text{def}}{=}\frac{1}{{\hat{\mu }}_{1}^{\left(k\right)}\left(1-{\hat{\mu }}_{1}^{\left(k\right)}\right)}$$

(37) $${\hat{\pi }}_{2}^{\left(k\right)}\overset{\text{def}}{=}\frac{1}{\sigma_{2}^{\left(k-1\right)}+t^{\left(k\right)}{\text{e}}^{\kappa \mu_{3}^{\left(k-1\right)}+\omega }}$$

Details of the derivation of these update equations are given in Appendix D. The update equations for binary outcomes differ from those given in Equations 9 and 10 only at the second level. On all higher levels, they are the generic HGF updates from Equations 9 and 10. This difference is entirely due to the sigmoid transformation that links the first and second level, enabling the filtering of binary outcomes. Note that in the binary case, the second level corresponds to the first level of the continuous case in the sense that they are the lowest levels where a Gaussian random walk takes place.

To illustrate of how the HGF can deal with the simplest kind of informational uncertainty, sensory uncertainty, we simulate two agents, one with high and the other with low sensory uncertainty, who are otherwise equal. Sensory uncertainty is captured by the following relation between the binary state *x*_1_ and sensory input *u*:

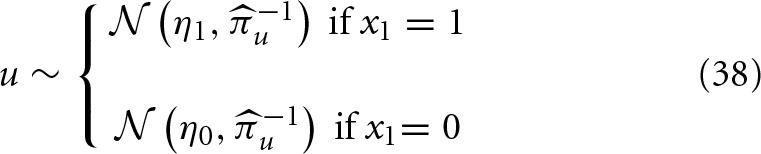



This means that the probability of *u* is Gaussian with precision $\hat{\pi }$_*u*_ around a mean of η_1_ for *x*_1_ = 1 and η_0_ for *x*_1_ = 0. In this case (cf. Mathys et al., 2011, Equation 47), the update equation for μ_1_ is

(39) $$\mu_{1}^{\left(k\right)}=\frac{{\hat{\mu }}_{1}^{\left(k\right)}\exp\left(-\frac{{\hat{\pi }}_{u}}{2}{\left(u^{\left(k\right)}-\eta_{1}\right)}^{2}\right)}{{\hat{\mu }}_{1}^{\left(k\right)}\exp\left(\text{​}-\frac{{\hat{\pi }}_{u}}{2}{\left(u^{\left(k\right)}-\eta_{1}\right)}^{2}\right)+\left(\text{​}1-{\hat{\mu }}_{1}^{\left(k\right)}\right)\exp\left(\text{​}-\frac{{\hat{\pi }}_{u}}{2}{\left(u^{\left(k\right)}-\eta_{0}\right)}^{2}\right)}$$

**Figure 3:**
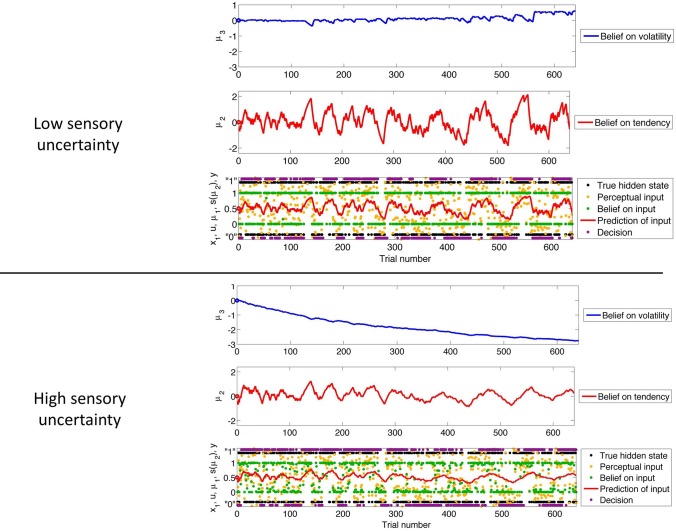
**The consequences of sensory uncertainty**. Simulation of inference on a binary hidden state *x*_1_ (black dots) using a three-level HGF under low ($\hat{\pi }$_*u*_ = 1000, top panel) and high ($\hat{\pi }$_*u*_ = 10, bottom panel) sensory uncertainty. Trajectories were simulated using the same input and parameters (except $\hat{\pi }$_*u*_) in both cases: μ^(0)^_2_ = μ^(0)^_3_ = 0, σ^(0)^_2_ = σ^(0)^_3_ = 1, κ = 1, ω = −3, and ϑ = 0.7. Decisions were simulated using a unit-square sigmoid model with ζ = 8.

Figure 3 shows our simulation. We first chose a sequence of true hidden states *x*^(*k*)^_1_, *k* = 1, …, 640. We then drew a sequence of inputs *u*^(*k*)^ ~ 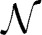 (*x*^(*k*)^_1_, 0.1), corresponding to a setting of η_1_ = 1, η_0_ = 0, and $\hat{\pi }$_*u*_ = 10 (cf. Equation 38). These inputs were fed into two three-level HGFs that differed only in the amount of sensory uncertainty they assumed: $\hat{\pi }$^−1^_*u*_ = 0.001 (low) and $\hat{\pi }$^−1^_*u*_ = 0.1 (high). Clearly, higher input precision leads to greater responsiveness to fluctuations in input, as reflected in the trajectory of belief on tendency *x*_2_ and in a higher volatility estimate (belief on *x*_3_). In the case of low input precision, the volatility estimate keeps declining because most of the variation in input is attributed to noise instead of fluctuations in the underlying tendency toward one outcome category *x*_1_ or the other. Decisions (purple dots) were simulated using a unit-square sigmoid response model with ζ = 8 in both cases (cf. Equation 18). The consequences of high sensory uncertainty for decision-making in this scenario are apparent: the agent with higher sensory uncertainty is less consistent in favoring one option over the other at any given time. This accords well with recent accounts of psychopathological symptoms as a failure of sensory attenuation (Adams et al., 2013).

We now turn to the third and fourth elements defining our Bayesian agent: decision models based on loss functions.

### Decision model for a simple binary loss function

One of the simplest decision situations for an agent is having to choose between two options, only one of which will be rewarded, but both of which offer the same gain (i.e., negative loss), if rewarded. In the three-level version of the HGF from Figure 2, we may code one such binary outcome as *x*_1_ = 1 and the other as *x*_1_ = 0. This allows us to define a quadratic loss function ℓ where making the wrong choice *y* ∈ {0, 1} leads to a loss of 1 while the right choice leads to a loss of 0:

(40) $$\ell (x_{1},y)={\left(x_{1}\text{-}y\right)}^{2}$$

The expected loss 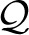 of decision *y*, given the agent's representations λ, is then the expectation of ℓ under the distributions *q* described by λ:

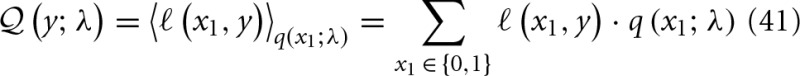



To evaluate this, we must remember that the agent has to rely on its beliefs deriving from time *k* − 1 to make decision *y*^(*k*)^ at time *k*. In the above equation, elements of λ therefore have time index *k* − 1, while *x*_1_ and *y* have time index *k*. Specifically, the belief on the outcome probability at the first level is $\hat{\mu }$^(*k*)^_1_ = *s* (μ^(*k*−1)^_2_). With *q*(*x*_1_) = (μ_1_)^*x*_1_^(1 − μ_1_)^1−*x*_1_^ (Mathys et al., 2011, Equation 12), we then have

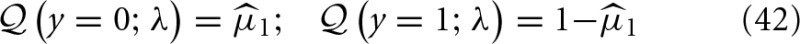



The optimal decision y^*^ is the one that minimizes expected loss 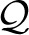:

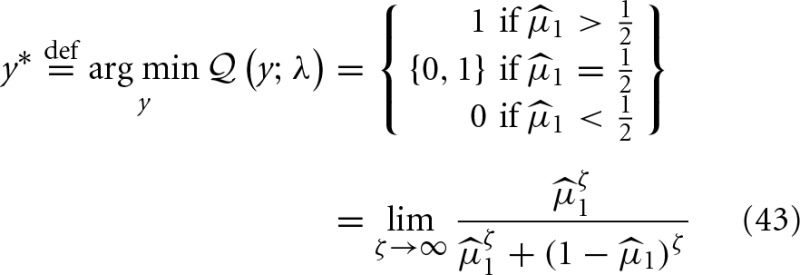



This simply means that to minimize its losses, the agent should choose the option it believes more likely to be rewarded. It may seem superfluous to go to such lengths to derive such an obvious result, but the purpose of the above is also to give an illustration of the principled way a decision rule can be derived by combining the HGF with a loss function.

It is, however, unreasonable to assume that human agents will always choose the option that minimizes their expected loss in the current trial, for two reasons. First, if there is more than one trial and the probabilities of the different options are independent, there is an exploration/exploitation tradeoff that makes it worth the agent's while (in the long run) sometimes to choose an option that is not expected to minimize loss in the current trial (Macready and Wolpert, 1998; Daw et al., 2006). Second, biological agents exhibit decision noise (Faisal et al., 2008), e.g., owing to implementation constraints at the molecular, synaptic or circuit level. To allow for exploration and noise, we use a decision model that corresponds to the right-hand side of Equation 43, without taking the limit, instead leaving ζ as a parameter to be estimated from the data (cf. Equation 18):

(44) $$p\left(y|\lambda ,\zeta \right)={\left(\frac{{\hat{\mu }}_{1}^{\zeta }}{{\hat{\mu }}_{1}^{\zeta }+{\left(1-{\hat{\mu }}_{1}\right)}^{\zeta }}\right)}^{y}\cdot {\left(\frac{{\left(1-{\hat{\mu }}_{1}\right)}^{\zeta }}{{\hat{\mu }}_{1}^{\zeta }+{\left(1-{\hat{\mu }}_{1}\right)}^{\zeta }}\right)}^{1-y}$$

**Figure 4:**
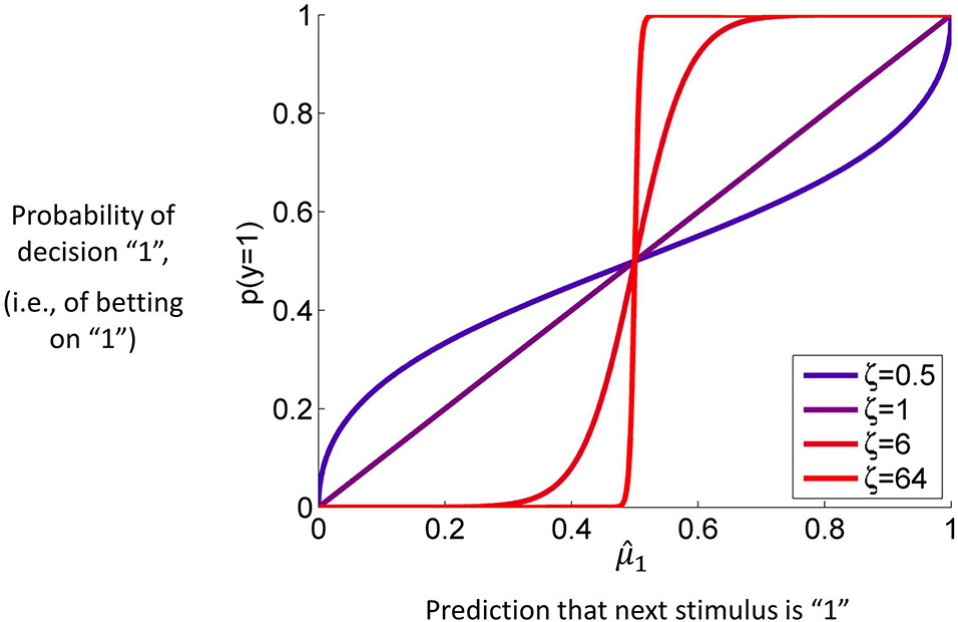
**The unit square sigmoid (cf. Equations 43, 44)**. The parameter ζ can be interpreted as inverse response noise because the sigmoid approaches a step function as ζ approaches infinity.

Figure 4 contains a graph of this function for *p*(*y* = 1) where ζ plays the role of the noise (or exploration) parameter. This decision model was the basis for the simulations we conducted to assess the accuracy of parameter estimations (results below).

### Inversion example

To illustrate how real datasets can be inverted and different response models compared, we take the data of one subject from Iglesias et al. (2013). This consists of 320 inputs *u* and responses *y*. Our perceptual model is the three-level HGF for binary outcomes without sensory noise, and a first choice of decision model is the unit-square sigmoid of Equation 44. Using the HGF Toolbox (http://www.translationalneuromodeling.com/tapas), we specify the following priors (mean, variance) in appropriately transformed spaces: μ^(0)^_2_: (0, 1), σ_2_: (0, 1), μ^(0)^_3_: (1, 0), σ^(0)^_3_: (0, 1), κ: (0, 2) ω: (−4, 0), ϑ: (0, 2), and ζ: (48, 1). The variance of 0 on μ^(0)^_3_ and ω fixes these parameters to 1 and −4, respectively. The spaces for σ^(0)^_i_ and ζ were log-transformed while κ was estimated in a logit-transformed space with upper bound 6, and ϑ was estimated in logit-transformed space with upper bound 0.005.

We now modify our response model so that it no longer has a constant free parameter (ζ) as its inverse decision temperature, but the inverse volatility estimate exp (− μ_3_):

(45) $$\begin{array}{l} p\left(y|\lambda \right)={\left(\frac{{\hat{\mu }}_{1}^{\exp\left(-\mu_{3}\right)}}{{\hat{\mu }}_{1}^{\exp\left(-\mu_{3}\right)}+{\left(1-{\hat{\mu }}_{1}\right)}^{\exp\left(-\mu_{3}\right)}}\right)}^{y}\cdot \\ \;{\left(\frac{{\left(1-{\hat{\mu }}_{1}\right)}^{\exp\left(-\mu_{3}\right)}}{{\hat{\mu }}_{1}^{\exp\left(-\mu_{3}\right)}+{\left(1-{\hat{\mu }}_{1}\right)}^{\exp\left(-\mu_{3}\right)}}\right)}^{1-y} \end{array}$$

**Figure 5:**
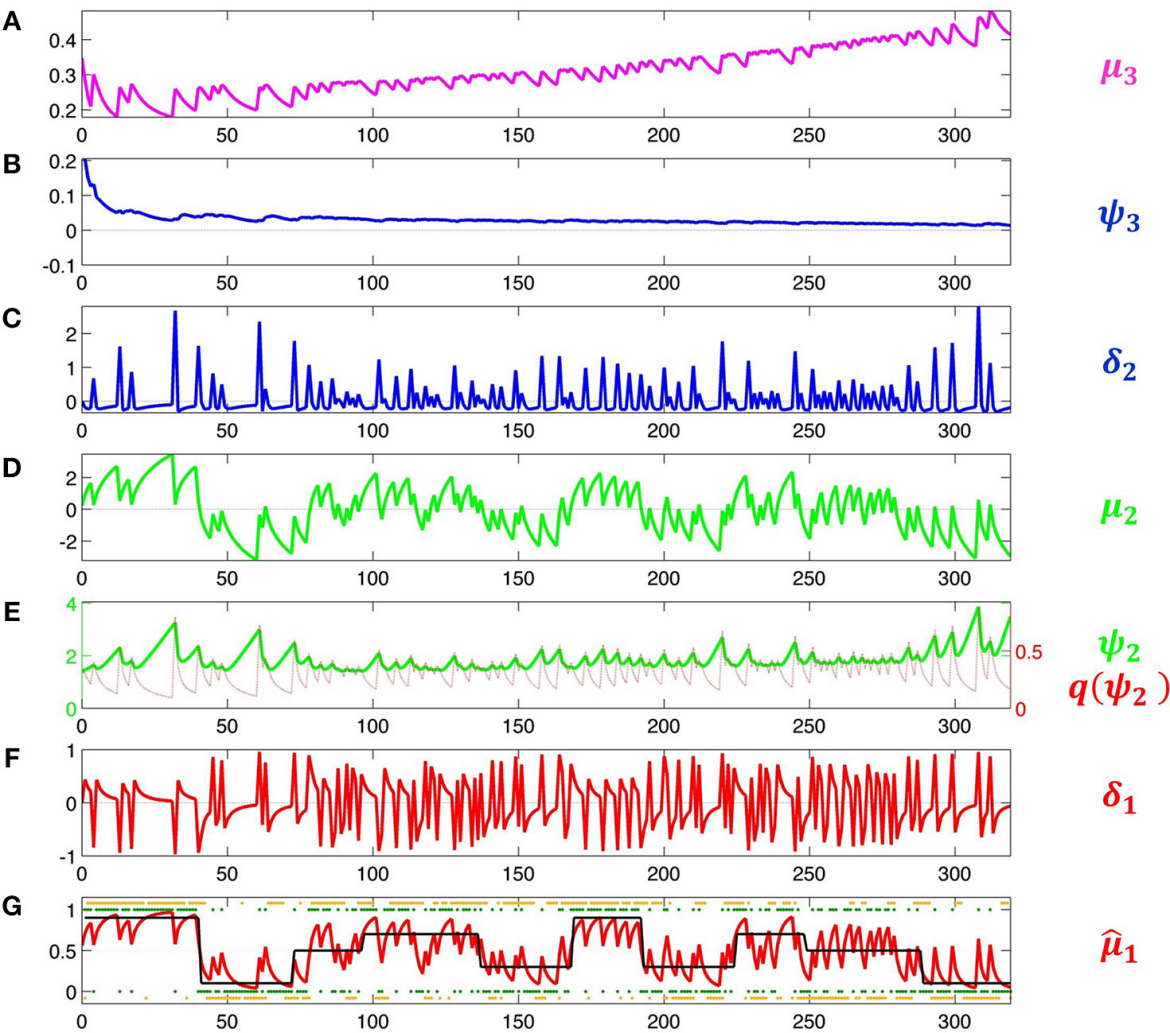
**Model inversion**. Maximum-a-posteriori parameter estimates are μ^(0)^_2_ = 0.87, σ^(0)^_2_ = 1.20, μ^(0)^_3_ = −0.65, σ^(0)^_3_ = 0.88, κ = 1.32, ω = −0.71, and ϑ = 0.0023. These parameter values correspond to the following trajectories: **(A)** Posterior expectation μ_3_ of log-volatility *x*_3_. **(B)** Precision weight $\psi_{3}\overset{\text{def}}{=}\frac{{\hat{\pi }}_{2}}{\pi_{3}}$ which modulates the impact of prediction error δ_2_ on log-volatility updates. **(C)** Volatility prediction error δ_2_. **(D)** Posterior expectation μ_2_ of tendency μ_2_. **(E)** Precision weight $\psi_{2}\overset{\text{def}}{=}\pi_{2}^{-1}$ (in green) which modulates the impact of input prediction error δ_1_ on μ_2_. Since μ_2_ is in logit space, the function of σ_2_ as a dynamic learning rate is more easily visible after transformation to *x*_1_-space. This results in the red line labeled $q\left(\psi_{2}\right)\overset{\text{def}}{=}s^{\prime }\left(\mu_{2}\right)\psi \_2\;)$. (F) Prediction error δ_1_ about input *u*. (In Iglesias et al., 2013, Figures S1 and S2, δ_1_ is defined as an outcome prediction error, which corresponds to the absolute value of δ_1_ as defined here). **(G)** Black: true probability of input 1. Red: posterior expectation of input *u* = 1, $\hat{\mu }$_1_; this corresponds to a sigmoid transformation of μ_2_ in **(E)**. Green: sensory input. Orange: subject's observed decisions.

This means that the agent will behave the less deterministically the more volatile it believes its environment to be. Since this is now a decision model that contains μ_3_, it permits us to estimate all parameters including μ^(0)^_3_ and ω. Accordingly, we increase the variance of their priors to 4. The result of this inversion is shown in Figure 5. This figure illustrates how the HGF deals with perceptual uncertainty by updating beliefs throughout its hierarchy on the basis of precision-weighted prediction errors. The learning rates ψ_*i*_ (definitions see figure caption) are adjusted continually at each level separately, which provides the flexibility needed to adapt to changes in outcome tendency and volatility (i.e., to perceptual uncertainty).

Under the Laplace assumption (Friston et al., 2007), the negative variational free energy, an approximation to the log-model evidence, is −196.19 for the first response model and −188.84 for the second. This corresponds to a Bayes factor of exp (−188.84 − (−196.19)) = 1556, giving the second model a decisive advantage despite the fact that it contains an additional free parameter. In this example, including a measure of higher-level uncertainty has clearly improved our model of a subject's learning and decision-making.

Another possible choice for the inverse decision temperature is $\hat{\pi }$_2_ (cf. Paliwal et al., 2014). This choice is interesting because it is similar to the hypothesis (Friston et al., 2013; Schwartenbeck et al., 2013) that precision serves as the inverse decision temperature in active inference. With a negative variational free energy of − 189.63, this model performs similarly to the one with exp (− μ_3_) as the inverse decision temperature. This similarity in performance is not surprising since $\hat{\pi }$_2_ is (inversely) driven to a large extent by μ_3_ (cf. Equations 11 and 13).

### Decision model for a one-armed bandit

**Figure 6:**
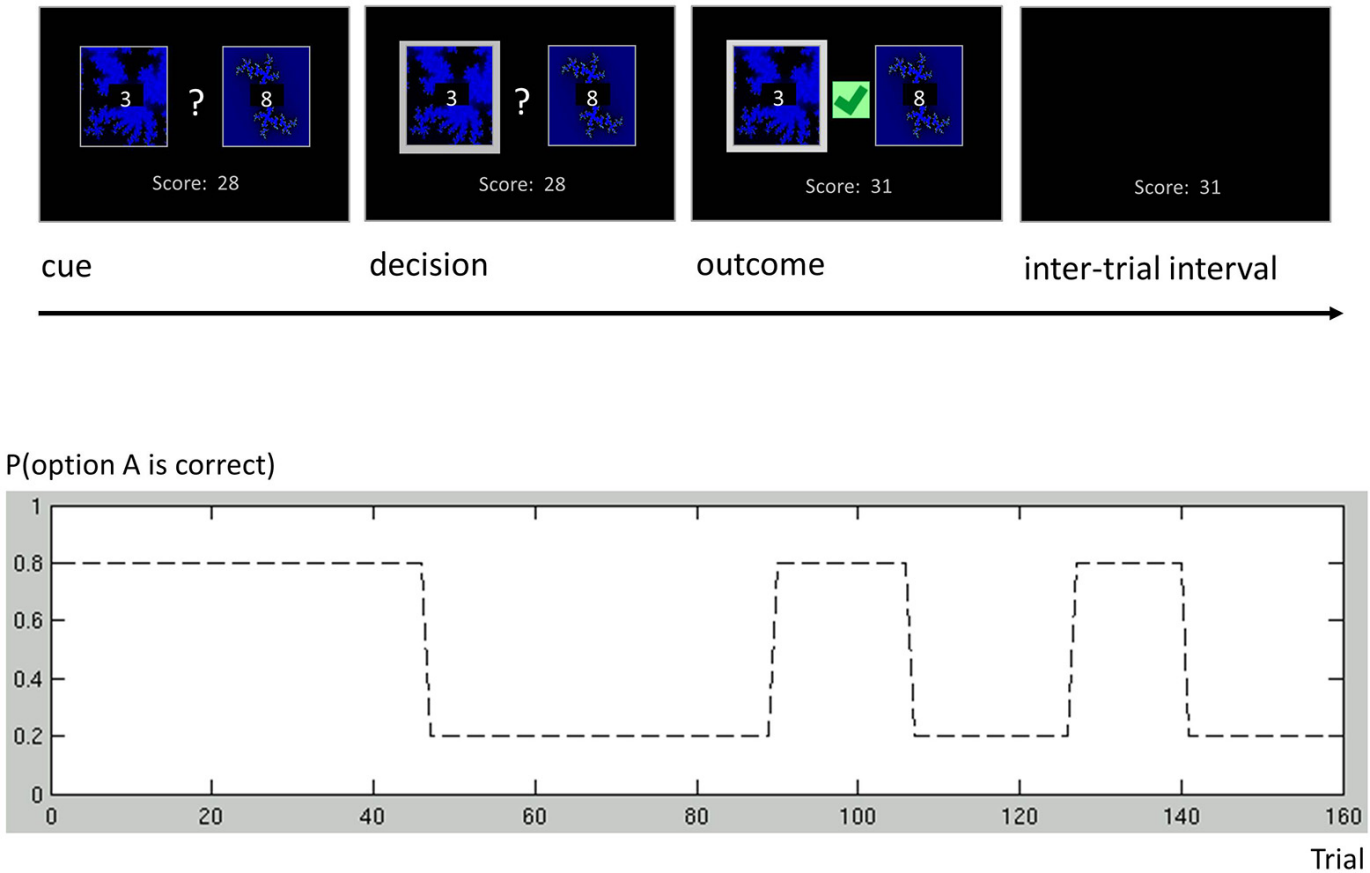
**One-armed bandit task**. Participants were engaged in a simple decision-making task. Each trial consisted of four phases. (i) Cue phase. Two cards and their costs were displayed. (ii) Decision phase. Once the subject had made a decision, the chosen card was highlighted. (iii) Outcome phase. The outcome of a decision was displayed, and added to the score bar only if the chosen card was rewarded. (iv) Inter-trial interval (ITI). The screen only showed the score bar, until the beginning of the next trial. Our experimental paradigm consisted of a number of phases with different reward structures. Different phase lengths induced both a phase of low volatility (trials 1 through 90) and a phase of high volatility (trials 91 through 160).

As an additional example, we discuss a more complex binary decision task that we used to collect data from human subjects (Cole et al., in preparation). In this variant of a one-armed bandit experiment, subjects were asked to play a series of gambles with the goal of maximizing their overall score (Figure 6). On each trial, subjects chose between two options represented by the same two fractals, which had different and time-varying reward probabilities. At any point in time, these probabilities summed to unity, implying that exactly one of the two options would be rewarded. Although subjects knew that probabilities varied throughout the course of the experiment, they were not told the schedule that governed these changes. The schedule included both a period of low volatility and a period of high volatility (Figure 6), similar to the task used by Behrens et al. (2007).

In order to encourage subjects to switch options above and beyond normal exploration behavior (Macready and Wolpert, 1998), the two cards were associated with varying reward magnitudes. On each trial, magnitudes were drawn from a discrete uniform distribution 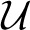
(1, 9) (i.e., rewards would take values from the range {1, 2,…, 9} with equal probability).

Subjects began the experiment with an initial score of 0 points. Once a card had been chosen, if that card was rewarded, the associated reward would be added to the current score. The final score at the end of the experiment was translated into monetary reimbursement. The experiment consisted of 160 trials.

Calling the two fractals A and B, we parameterize the agent's response by

(46) $$y=\{\begin{array}{c} 0\text{ for choice A}\\ 1\text{ for choice B} \end{array}$$

Correspondingly, the state *x*_1_ is

(47) $$x_{1}=\{\begin{array}{c} 0\text{ if A rewarded}\\ 1\text{ if B rewarded} \end{array}$$

Taking *r_A_* and *r_B_* to be the rewards for A and B, respectively, we introduce the quadratic loss function

(48) $$\begin{array}{l} \ell (x,y)=-(1-x_{1}){\left(y+x_{1}-1\right)}^{2}\cdot r_{A}-x_{1}{\left(y+x_{1}-1\right)}^{2}\cdot r_{B}\\ \;=-{\left(y+x_{1}-1\right)}^{2}(\left(r_{B}-r_{A}\right)x_{1}+r_{A}) \end{array}$$

This corresponds to the following loss table:

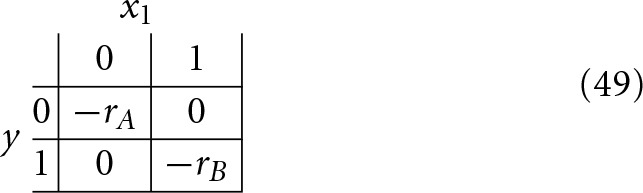



Following the same procedure as above, we get:

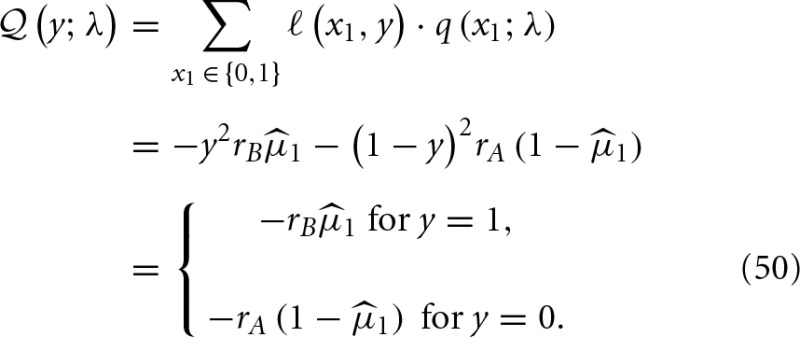



With the expected loss from each option on a continuous scale, a simple but powerful decision model is the softmax rule (Sutton and Barto, 1998; Daw et al., 2006)

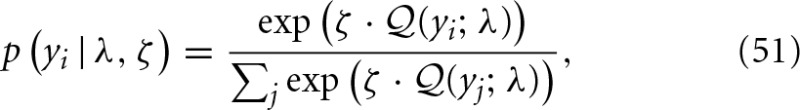



where *y_i_* is one particular option and the sum runs over all options. This means that the decision probabilities are Boltzmann-distributed according to their expected rewards (i.e., their expected negative losses) with the parameter ζ serving as the analogon of inverse temperature. In our binary case, this evaluates to

(52) $$\begin{array}{l} p\left(y=1|\lambda ,\zeta \right)=s(-\zeta (r_{B}{\hat{\mu }}_{1-}r_{A}(1-{\hat{\mu }}_{1}))),\\ p\left(y=0|\lambda ,\zeta \right)=s(+\zeta (r_{B}{\hat{\mu }}_{1}-r_{A}(1-{\hat{\mu }}_{1}))). \end{array}$$

This is a logistic sigmoid function of the difference *r_B_* $\hat{\mu }$_1_ − *r_A_* (1 − $\hat{\mu }$_1_) of expected reward for choice B minus expected reward for choice A. If the expected reward of choice B exceeds that of choice A, the likelihood of choice B is greater than half and vice versa.

### Simulation study

Given the nontrivial nature of our model, it is important to verify the robustness of the inversion scheme. This, in turn, may depend on the numerical optimization method employed. To assess our ability to estimate the parameters under different optimization schemes, we conducted a systematic simulation study based on the 3-level HGF for binary outcomes. This model is shown graphically in Figure 2 and was the basis for the studies of Vossel et al. (2013) and Iglesias et al. (2013). κ was chosen as the perceptual parameter to vary because of the interesting effects it has on the nature of the inferential process (cf. Mathys et al., 2011). The response parameter ζ was chosen as the second parameter to vary because it represents inverse response noise (cf. Equation 20), i.e., for lower values of ζ the mapping from beliefs to responses becomes less deterministic, which renders it more difficult to estimate the perceptual parameters.

Simulations took place in four steps:

1. We chose a particular sequence of 320 binary input *u* = {*u*^(1)^, …, *u*^(320)^}; this was the input sequence in a recent study using the HGF (Iglesias et al., 2013).
2. We chose a particular set of values for the parameters ξ.
3. We generated 320 binary responses *y* = {*y*^(1)^, …, *y*^(320)^} by drawing from the response distribution given by Equation 44 below.
4. We estimated ξ^*^ according to Equation 21.

Step 1 was only performed once, so that *u* was the same in all simulations. The values of ξ in step 2 were constant for all parameters except κ and ζ. The values of κ and ζ were taken from a two-dimensional grid in which the κ dimension took the values {0.5, 1, 1.5, …, 3.5} while the ζ dimension took the values {0.5, 1, 6, 24}. Steps 3 and 4 were then repeated 1'000 times for each value pair on the {κ, ζ} grid (for MCMC, owing to its computational burden, only 100 estimations were performed). The ζ values on the grid were chosen such that they covered the whole range from very low (ζ = 24) to very high response noise (ζ = 0.5, cf. Figure 4). The κ values were chosen to cover the range observed in an empirical behavioral study using the same inputs *u* (Iglesias et al., 2013). The remaining model parameters were held constant (ω = −4, ϑ = 0.0025). In total, six parameters were estimated. These were (with the space they were estimated in and prior mean and variance in that space in brackets): μ^(0)^_2_ (native, 0, 1), σ^(0)^_2_ (log, 0, 1), σ^(0)^_3_ (log, 0, 1), κ (logit with upper bound at 6, 0, 9), ϑ (logit with upper bound at 0.005, 0, 9), and ζ (log, 48, 1). The prior mean of the response variable ζ was chosen relatively high to provide shrinkage on the estimation of decision noise.

This procedure was repeated for four different optimization methods which are commonly used but possess different properties with regard to computational efficiency and robustness to getting trapped in local extrema:

1. Nelder-Mead simplex algorithm (NMSA),
2. Gaussian process-based global optimization (GPGO),
3. Variational Bayes (VB),
4. Markov Chain Monte Carlo estimation (MCMC).

In brief, NMSA (Nelder and Mead, 1965) is a popular local optimization algorithm which is implemented, for example, in the fminsearch function of Matlab. VB also optimizes locally (by gradient descent); for details see Bishop (2006, p. 461ff). For our simulation study, we used VB as implemented in the DAVB toolbox, available at http://goo.gl/As8p7 (Daunizeau et al., 2009, 2014). In contrast, GPGO (Rasmussen and Williams, 2006; Lomakina et al., 2012) provides a global optimum of the objective function and is thus potentially more robust than NMSA and VB albeit computationally more expensive. The final method was MCMC (Gelman et al., 2003, p. 283ff) which served as a “gold standard” against which we compared the other methods. Specifically, we used Gibbs sampling with a one-dimensional Metropolis step for each of the parameters (cf. Gelman et al., 2003, p. 292). For each of the 100 simulation runs (at each point on our parameter grid) we used one chain with a length of 500'000 samples and a burn-in period of 25'000 samples. In summary, our simulations thus consider two algorithms (NMSA and VB) which are computationally very efficient but provide a local optimum only, in comparison to another two algorithms (GPGO and MCMC) which are computationally more expensive but are capable of finding global optima.

**Figure 7:**
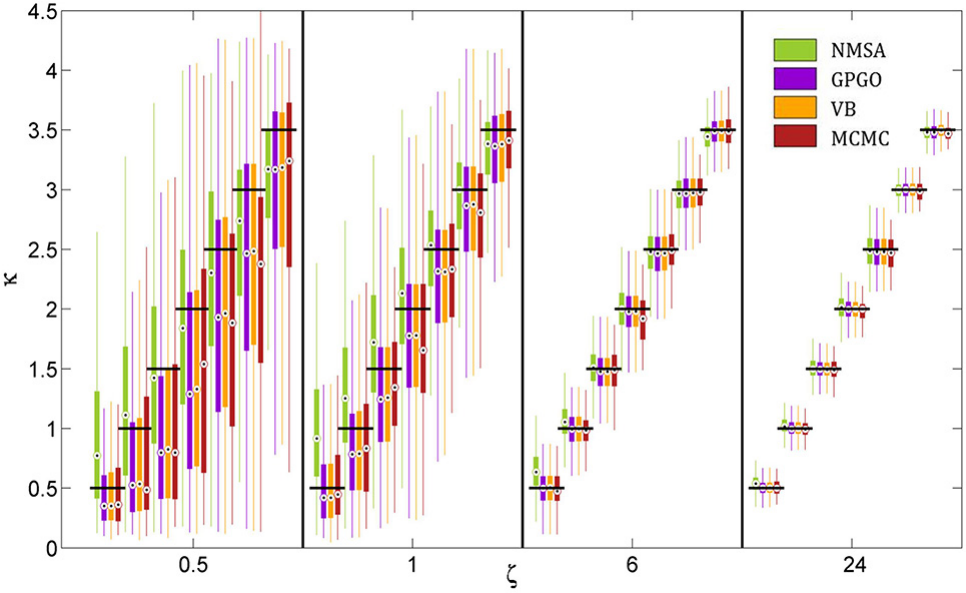
**Estimation of coupling κ by four methods at different noise levels ζ**. A range of κ from 0.5 to 3.5 was chosen based on the range of estimates observed in the analysis of experimental data. Decision noise levels were chosen in a range from very high (0.5) to very low (24). The remaining model parameters were held constant (ω = −4, ϑ = 0.0025). For each point of the resulting two-dimensional grid, 1000 task runs with 320 decisions each were simulated. Given the fixed sequence of inputs and simulated sequence of decisions, we then attempted to recover the model parameters, including κ and ζ, by four estimation methods: (1) the function Nelder-Mead simplex algorithm (NMSA), (2) Bayesian global optimization based on Gaussian processes (GPGO), (4) variational Bayes (VB), and Markov chain Monte Carlo sampling (MCMC). The figure shows boxplots of the distributions of the maximum-a-posteriori (MAP) point estimates for the four methods at each grid point. Boxplots consist of boxes spanning the range from the 25th to the 75th percentile, circles at the median, and whiskers spanning the rest of the estimate range. Horizontal shifts within ζ levels are for readability. Black bars indicate ground truth.

All optimization methods could reliably distinguish different values of κ at low or moderate decision noise (Figure 7). At higher noise levels, estimates became less reliable. With GP, VB, and MCMC, they then exhibited a tendency to underestimate κ, while NMSA tended to mid-range values. Nonetheless, substantial differences in κ within the range tested could be detected by all four methods even at high levels of noise.

**Figure 8:**
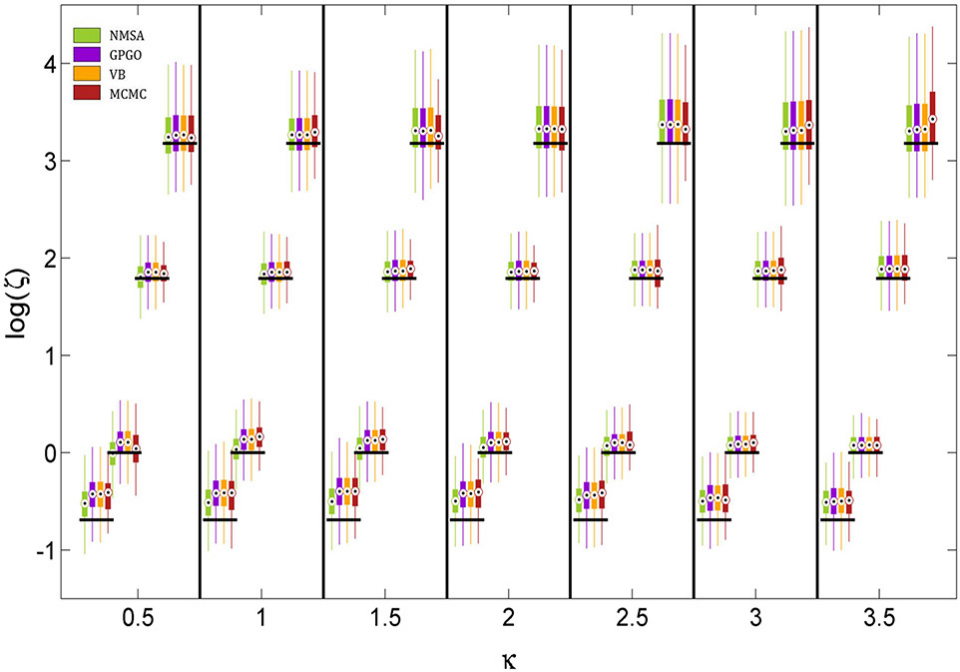
**Estimation of noise level ζ at different levels of coupling κ**. ζ is estimated and displayed here at the logarithmic scale because it has a natural lower bound at 0. See Figure 7 for key to legend. The figure shows boxplots of the distributions of the maximum-a-posteriori (MAP) point estimates for the four methods at each point of the simulation grid. Horizontal shifts within κ levels are for readability. Black bars indicate ground truth.

**Figure 9:**
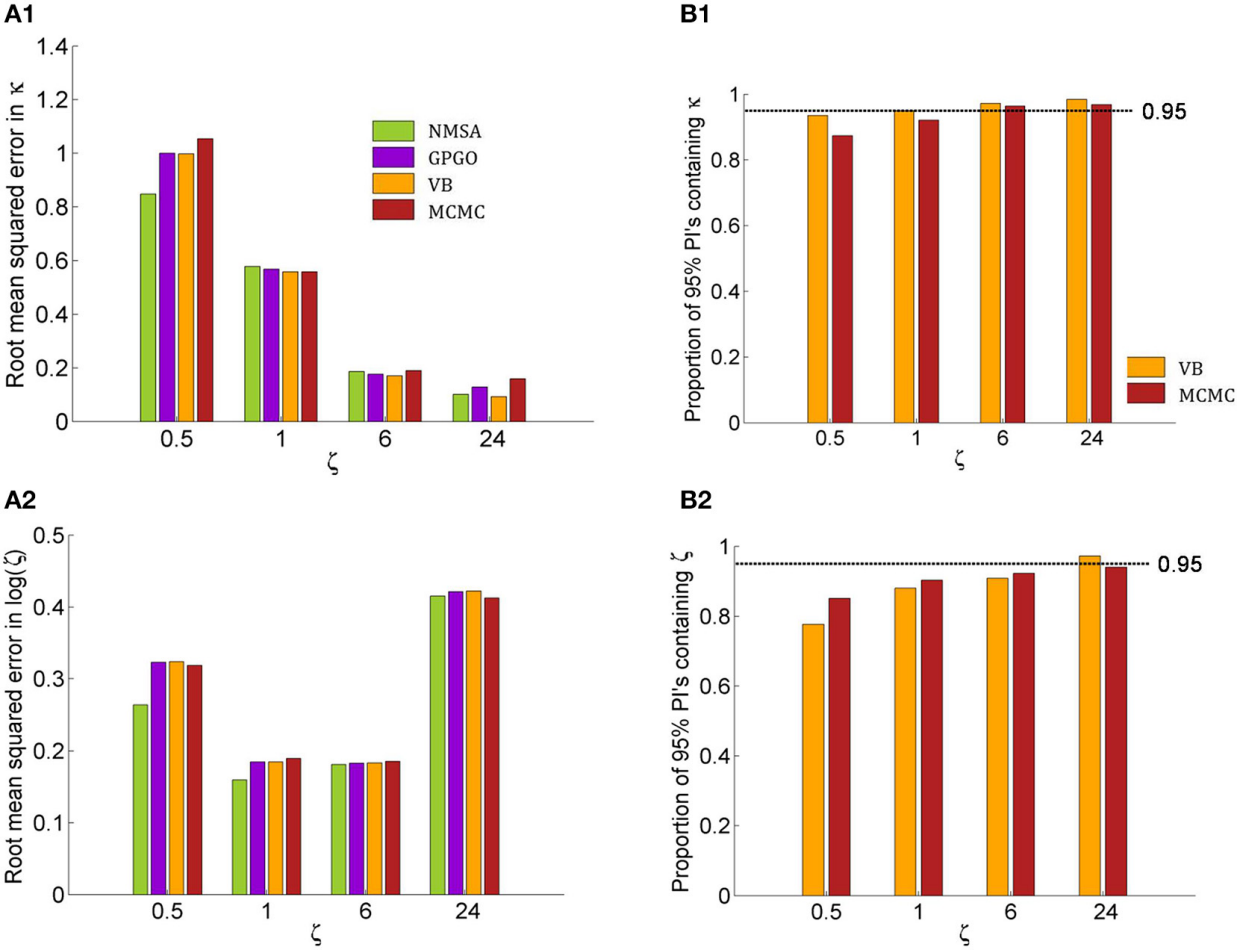
**Quantitative assessment of parameter estimation**. **(A)** Root mean squared error of MAP estimates by noise level ζ for all four estimation methods (see Figure 7 for key to legends). **(A1)** Estimates for κ improve with decreasing noise and do not exhibit substantially significant differences between methods although NMSA is somewhat better at very high noise. **(A2)** As in Figure 8, estimates for ζ were assessed at the logarithmic scale. **(B)** Confidence of VB and MCMC. **(B1)** Both methods are realistically confident about their inference on κ across noise levels, with a slight tendency toward overconfidence with higher noise. **(B2)** This tendency is more pronounced with estimates of ζ.

The noise level itself could also be determined by all four methods (Figure 8). The methods did not differ appreciably in their performance. They all tended to underestimate the noise level owing to a mild shrinkage due to the prior on ζ. Errors are smaller for moderate noise levels, increasing for both high and low noise (cf. Figure 9A2).

Figures 9A1,A2 shows the root mean squared error in κ and log (ζ), jointly for all values of κ. The results show that the noise level could best be estimated at moderate levels, where in fact most estimates of experimental data are found. Again, the methods perform comparably well, with NMSA best at high noise. Figures 9B,B2 contrasts the performance of VB and MCMC and displays the accuracy of the confidence with which VB and MCMC make their estimates. To this end, it uses the fact that VB and MCMC estimate the whole posterior distribution. Parameter estimates can therefore not only be summarized as point estimates, but also as posterior central intervals (PCIs; the 95% PCI is the interval that excludes 2.5% of the posterior probability mass on either side). If an estimation method were neither over- nor underconfident, 95 of 95% PCIs would contain the true parameter value. If the proportion is less than 0.95, this indicates overconfidence; if it is greater than 0.95, underconfidence. Both methods were realistically confident about their inference on κ across noise levels, with a slight tendency toward overconfidence with higher noise. This tendency was more pronounced with estimates of ζ.

## Discussion

In this paper, we have shown that the hierarchical Bayesian model of Mathys et al. (2011) can be extended in several ways, resulting in a general framework referred to as the HGF. Furthermore, we have demonstrated how the HGF can be combined with decision models to allow for parameter estimation from empirical data. We start by discussing the nature of the HGF updates in the context of Bayesian inference.

A crucial feature of the HGF's update equations is emphasized by the notation used in Equation 9: the updates of the means are *precision-weighted prediction errors*. For a full understanding of their role, we will first discuss Bayesian updates in the simplest possible case, where they can be calculated exactly. In this simplest case, there is only one hidden state *x* ∈ ℝ that is the target of our inference, and there is a Gaussian prior on *x*:

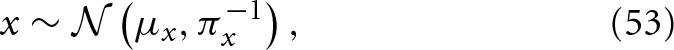



where μ_*x*_ is the mean and π_*x*_ the precision. The likelihood of *x* (i.e., the probability of observing the datum *u* ∈ ℝ given *x*) is also Gaussian, with precision (inverse observation noise) π_*u*_:

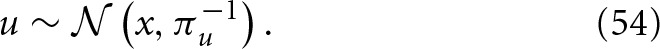



According to Bayes' theorem, the posterior is now also Gaussian:

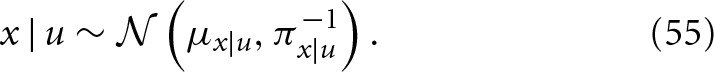



The posterior precision π_*x*|*u*_ and mean μ_*x*|*u*_ can be written as the following analytical and exact one-step updates:

(56) $$\mu_{x|u}\;=\mu_{x}+\frac{\pi_{u}}{\pi_{x|u}}\left(u-\mu_{x}\right)$$

(57) $$\pi_{x|u}=\pi_{x}+\pi_{u}$$

The update in the mean is a precision-weighted prediction error. The prediction error *u* − μ_*x*_ is weighted proportionally to the observation precision π_*u*_, reflecting the fact that the more observation noise there is, the less weight should be assigned to the prediction error. On the other hand, prediction error is weighted inversely proportionally to the posterior precision π_*x*|*u*_; that is, with higher certainty about *x*, the impact of any new information on its estimate becomes smaller.

**Figure 10:**
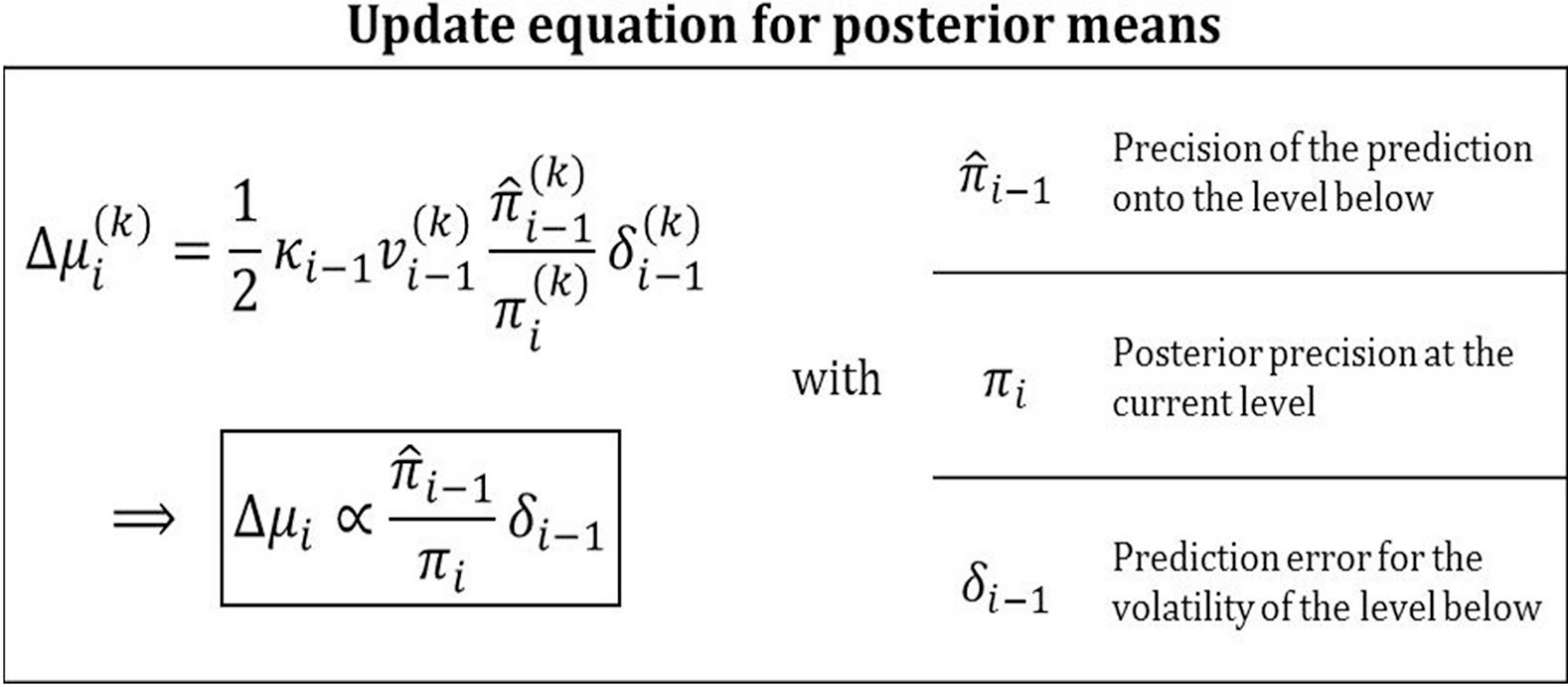
**Posterior mean update equation**. Updates are precision-weighted prediction errors. This general feature of Bayesian updating is concretized by the HGF for volatility predictions in a hierarchical setting.

The same precision-weighting of prediction errors appears in the update of the means μ_*i*_ of the states *x*_*i*_ in the inversion of the general HGF (Figure 10, Equation 9):

(58) $$\mu_{i}^{\left(k\right)}-{\hat{\mu }}_{i}^{\left(k\right)}=\frac{1}{2}\kappa_{i-1}v_{i-1}^{\left(k\right)}\frac{{\hat{\pi }}_{i-1}^{\left(k\right)}}{\pi_{i}^{\left(k\right)}}\delta_{i-1}^{\left(k\right)},$$

or, in more compact notation,

(59) $$\Delta \mu_{i}\propto \frac{{\hat{\pi }}_{i-1}}{\pi_{i}}\delta_{i-1}.$$

Owing to the hierarchical nature of the HGF, the place of the likelihood precision π_*u*_ in Equation 56 is here taken by the precision of the prediction on the level below, $\hat{\pi }$_*i*_, while the posterior precision π_*i*_ in the HGF corresponds exactly to the posterior precision π_*x*|*u*_ in Equation 56. The precision ratio in these updates is the *learning rate* with which the prediction error is weighted. Prediction errors weighted by a learning rate are a defining feature of many reinforcement learning models (e.g., Rescorla and Wagner, 1972). The HGF furnishes a Bayesian foundation for these heuristically derived models in that it provides learning rates that are optimal given a particular agent's parameter setting. The numerator of the precision ratio in Equation 59 contains the precision of the prediction onto the level below. This relation make sense because the higher this precision, the more meaning a given prediction error has. The denominator of the ratio contains the precision of the belief about the level being updated. Again, it makes sense that the update should be antiproportional to this since the more certain the agent is that it knows the true value of *x_i_*, the less inclined it should be to change it. What sets the HGF apart from other models with adaptive learning rates (e.g., Sutton, 1992; Nassar et al., 2010; Payzan-LeNestour and Bossaerts, 2011; Wilson et al., 2013) is that its update equations are derived to optimize a clearly defined objective function, variational free energy, thereby minimizing surprise. Furthermore, while the Kalman filter (Kalman, 1960) is optimal for data generated by linear dynamical systems, the HGF has the advantage that it can deal with nonlinear systems because it adapts its volatility estimate as the data come in. This adaptive adjustment of learning rates corresponds to an optimal “forgetting” algorithm that prevents learning rates from becoming too low.

Notably, the prediction error δ_*i* − 1_ is a volatility prediction error (VOPE) in the HGF while the prediction errors in the single-level Gaussian updates Equation 56, like the first-level updates in the HGF Equation 15, refer to value prediction errors (VAPEs). While a VAPE captures the error about the magnitude of a hidden state, a VOPE captures the error about the amount of change in a hidden state. The crucial point here is that the levels of the HGF are linked via the variance (or, equivalently, precision) of the prediction of the next lower level. Consequently, the inversion proceeds by updating the higher level based on the variance (or volatility) of the lower level. This becomes apparent in Equation 14. The denominator of the fraction contains predicted uncertainty about the level below, while the numerator contains observed uncertainty. These can again be broken down into informational and environmental uncertainty (see below). Whenever observed uncertainty exceeds predicted, the fraction is greater than one and the VOPE is positive. Conversely, when observed uncertainty is less than predicted, the VOPE is negative.

The two sources of uncertainty, informational and environmental, are clearly visible in the precision of the predictions Equation 13 and in the VOPEs Equation 14. In Equations 13 and 14, σ^(*k* − 1)^_*i*_ is the informational posterior uncertainty about *x*_*i*_, while $v_{i}^{\left(k\right)}=t^{\left(k\right)}\exp\left(\kappa_{i}\mu_{i\;+\;1}^{\left(k-1\right)}+\omega_{i}\right)$ is the environmental uncertainty, the magnitude of which is determined by a combination of two kinds of volatility: phasic $\left(\mu_{i\;+\;1}^{\left(k-1\right)}\right)$ and tonic (ω_*i*_). The less we know about *x_i_*, the greater the informational uncertainty σ^(*k* − 1)^_i_; by contrast, the more volatile the environment is, the greater the environmental uncertainty *v*^(*k*)^_*i*_.

The relation between uncertainty (informational and environmental, expected and unexpected) and volatility (phasic and tonic) can be summarized as follows: informational uncertainty could be seen as a form of expected uncertainty which, however, differs from Yu and Dayan (2005) in that it is defined in terms of posterior variance instead of estimated deviation from certainty. By constrast, environmental uncertainty can be linked to “unexpected” uncertainty and is the result of phasic and tonic volatility. We use the term “environmental” instead of “unexpected” because, in the context of the HGF, unexpected uncertainty is incorporated into the precision of predictions (cf. Equation 13), i.e., there is always some degree of belief that the environment might be changing.

In a review of the literature on different kinds of uncertainty in human decision-making, Bland and Schaefer (2012) argue that unexpected uncertainty and volatility are often not sufficiently differentiated while Payzan-LeNestour and Bossaerts (2011) make a further subdistinction of unexpected uncertainty: they differentiate between stochastic volatility and a narrower concept of unexpected uncertainty. This distinction maps exactly onto the difference between tonic and phasic volatility in the HGF. While the Kalman Filter deals optimally with tonic/stochastic volatility, the HGF can also accommodate sudden environmental changes via phasic volatility. An illustration of this ability can be found in Mathys et al. (2011), where the U.S. Dollar to Swiss Franc exchange rate time series from the first half of the year 2010 is filtered using the HGF (their Figure 11). In addition to tonic volatility, this time series also reflects a clear change point, i.e., the markets' realization that Greece was insolvent. The latter is captured by the HGF in phasic volatility shooting up almost vertically.

Environmental uncertainty is updated by adjusting μ_*i* + 1_, the estimate of the next higher level. This is done in the VOPE by comparing predicted total uncertainty (informational plus environmental, σ^(*k* − 1)^_*i*_ + *v*^(*k*)^_*i*_) to observed total uncertainty $\left(\sigma_{i}^{\left(k\right)}+{\left(\mu_{i}^{\left(k\right)}-{\hat{\mu }}_{i}^{\left(k\right)}\right)}^{2}\right)$. In this way, environmental uncertainty estimates are dynamically adapted to changes in the environment, leading to changes in learning rates that reflect an optimal (with respect to avoiding surprise) balance between informational and environmental uncertainty estimates.

At first glance, the precision-weighting of prediction errors may seem different in the “classical” 3-level HGF (Figure 2) with categorical outcomes, where the update for μ_2_ (Equation 32) is:

(60) $$\mu_{2}^{\left(k\right)}=\mu_{2}^{\left(k-1\right)}+\sigma_{2}^{\left(k\right)}\delta_{1}^{\left(k\right)}$$

At first, this simply looks like an uncertainty-weighted (not precision-weighted) update. However, if we unpack σ_2_ according to Equation 33 and do a Taylor expansion in powers of $\hat{\pi }$_1_, we see that it is again proportional to the precision of the prediction on the level below:

(61) $$\begin{array}{l} \sigma_{2}^{\left(k\right)}=\frac{{\hat{\pi }}_{1}^{\left(k\right)}}{{\hat{\pi }}_{2}^{\left(k\right)}{\hat{\pi }}_{1}^{\left(k\right)}+1}={\hat{\pi }}_{1}^{\left(k\right)}\text{-}{\hat{\pi }}_{2}^{\left(k\right)}{\left({\hat{\pi }}_{1}^{\left(k\right)}\right)}^{2}+{\left({\hat{\pi }}_{2}^{\left(k\right)}\right)}^{2}{\left({\hat{\pi }}_{1}^{\left(k\right)}\right)}^{3}\\ \text{ +}O\text{(4).} \end{array}$$

We have further shown a principled way how to define decision models based on perceptual HGF inferences, namely by deriving them from a loss function. Based on such decision models, it is possible to infer on model parameters and state trajectories from observed decisions. In the simulation study we have reported here, we could show that even with considerable decision noise, we can reliably infer model parameters based on a few hundred data points for binary decisions. Several recent studies have done this in practice, estimating subject-specific HGF parameters from behavioral data. For example, (Vossel et al., 2013) used the HGF to model learning in human subjects performing a Posner task with varying outcome contingencies. This study sought to compare different possible explanations for measured eye movements (saccadic reaction speeds), using a factorial model space comprising three alternative perceptual and three different response models. Model comparison showed that the 3-level HGF had greater model evidence than simpler versions of itself and Rescorla–Wagner learning (Rescorla and Wagner, 1972). This indicates that humans are capable of hierarchically structured learning, exploiting volatility estimates to adapt their learning rate dynamically. The same conclusion emerged from the study of Iglesias et al. (2013) who used the HGF to analyze human learning of auditory-visual associations which varied unpredictably in time. This study subsequently used the trial-wise estimates of precision-weighted prediction errors (i.e., $\frac{{\hat{\pi }}_{i-1}}{\pi_{i}}\delta_{i-1}$) in fMRI analyses, demonstrating activation of the dopaminergic midbrain with first-level (i.e., sensory outcome) precision-weighted prediction errors, and activation of the cholinergic basal forebrain with second-level (i.e., probability) precision-weighted prediction errors. These findings resonate with recent proposals that an important aspect of neuromodulatory function is the encoding of precision (Friston, 2009).

The HGF can, in principle, accommodate any form of loss function in the decision model. This choice will depend on the particular question addressed and the assumptions of the application domain (e.g., rationality assumptions). In the examples shown in this paper, we employ loss functions that are quadratic. This reflects the fact that squared losses imply a Gaussian distribution of errors, which is the appropriate choice where little is known about the true distribution because the Gaussian has maximum entropy (i.e., the least arbitrary assumptions) for a given mean and variance. This means that using a quadratic loss function is the most conservative choice in the absence of additional prior knowledge about the error distribution, where the term error refers to the agent's failure to make the choice that minimizes its expected loss.

Since all four methods of inverting the decision model performed well in our simulation study, we may focus on secondary criteria in choosing a method for practical applications. The most important of these criteria are the computational burden imposed and the amount of information contained in the estimate. The best performer in these respects is currently variational Bayes because it is efficient and provides an estimate of the whole posterior distribution for all parameters in addition to an approximation to the free energy bound on the log-model evidence, enabling model comparison. MCMC offers the same in principle, but at a considerably higher computational cost. GPGO is computationally more expensive than VB but may be a strong contender for future cases with multimodal posterior distributions. The weakest contender is NMSA because it is not much more efficient than VB but only offers a point estimate of the MAP parameter values.

In summary, the HGF provides a general and powerful framework for inferring on belief updating processes and learning styles of individual subjects in a volatile environment. This makes it a generic tool for studying perception in a Helmholtzian sense. The simple nature of the HGF updates in the form of precision-weighted prediction errors do not only enhance their biological interpretability and plausibility (cf. Friston, 2009) but are also crucial for practical applications. The ability of the HGF to infer learning styles of individual subjects from behavioral data and its support of Bayesian model comparison offer interesting opportunities for studying individual differences and particularly for clinical studies on psychopathology. To facilitate such practical applications, we have developed a software toolbox based on Matlab that is freely available for downloading as part of the TAPAS collection at http://www.translationalneuromodeling.org/tapas/. The HGF toolbox is specifically tailored to the implementations of discrete time filtering models, as opposed to the DAVB toolbox, which is mostly aimed at inverting dynamic models in continuous time. The HGF toolbox implements most of the models described in this article, plus some additional ones, and will be the focus of a forthcoming article.

### Conflict of interest statement

The authors declare that the research was conducted in the absence of any commercial or financial relationships that could be construed as a potential conflict of interest.

## Appendices

### (A) coupling between levels

Since *f* (*x*) is a positive function, there must be a function *g* (*x*) whose exponential it is.

(A1) f(x)>0 ∀ x ⇒ ∃ g:f(x)=exp(g(x)) ∀ x

Expanding *g*(*x*) then amounts to expanding the logarithm of *f*(*x*).

(A2) $$\begin{array}{l} g\left(x\right)=g\left(a\right)+g^{\prime }\left(a\right)\cdot \left(x-a\right)+O\left(2\right)=\log f\left(x\right)=\\ \;=\log f\left(a\right)+\frac{f^{\prime }(a)}{f(a)}\cdot \left(x-a\right)+O(2)=\\ \;=\underset{\overset{\text{def}}{=}\kappa }{\underset{︸}{\frac{f^{\prime }(a)}{f(a)}}}\cdot \;x+\underset{\overset{\text{def}}{=}\omega }{\underset{︸}{\log f\left(a\right)-a\cdot \frac{f^{\prime }\left(a\right)}{f\left(a\right)}}}+O\left(2\right)=\\ \;=\kappa x+\omega +O\left(2\right) \end{array}$$

(A3) ⇒f(x)≈exp(κx+ω)

Given *f*(*x*), κ and ω are only unique with respect to the choice of a particular expansion point *a*, except when *f*(*x*) is the exponential of a first-order polynomial, in which case the above approximation is exact. The greater the weight of higher-order terms in *g*(*x*), the less accurate the approximation will be far from the expansion point *a*. In ignoring second and higher order terms, we effectively restrict the HGF to first-order coupling functions (i.e., to coupling functions that are the exponentials of first-order polynomials). However, as this derivation shows, locally (i.e., within small variations of *x*), this restriction does not matter.

### (B) variational inversion of the HGF

Variational inversion provides closed-form one-step update equations for the sufficient statistics that describe the agent's belief about the state of its environment. These update equations are derived in two steps. First, for a particular level *i*, 1 < *i* < *n*, of the hierarchy, a mean field approximation is introduced, where the distributions *q*(*x*_*j*_) for all the other levels *j* ≠ *i* are assumed to be known and Gaussian, fully described by the known sufficient statistics {μ_*j*_, σ_*j*_}_*j*≠*i*_:

In the absence of sensory noise, *x*_1_ is directly observed; this means that μ^(*k*)^_1_ = *x*^(*k*)^_1_ and σ^(*k*)^_1_ = 0 for all *k*. The approximate posterior $\hat{q}$(*x*^(*k*)^_*i*_) for level *i* at time *k*, given sensory input μ^(1 … *k*)^_1_ = {μ^(1)^_1_, μ^(2)^_1_, …, μ^(*k*)^_1_} is then

with variational energy *I*,

(B3) $$\begin{array}{l} I\left(x_{i}^{\left(k\right)}\right)=\int q\left(x_{\backslash i}^{\left(k\right)}\right)\ln p\left(x_{i}^{\left(k\right)},x_{\backslash i}^{\left(k\right)},\chi |\mu_{1}^{\left(1\ldots k\right)}\right)\text{d}x_{\backslash i}^{\left(k\right)},\\ q\left(x_{\backslash i}^{\left(k\right)}\right)=\prod_{j\neq i}q\left(x_{j}\right),\\ \;x_{\backslash i}={\left\{x_{j}\right\}}_{j\neq i},\\ \;\chi ={\left\{\kappa_{i},\omega_{i},\vartheta \right\}}_{1<i<n}. \end{array}$$

We further unpack this to get

Performing this last integral, we obtain

This constitutes the “prediction step” of the update. Specifically, we let the random walk do its work by integrating out all states from the previous time point, thus ensuring that the mean field approximation only applies to current values of states. This is akin to the prediction step in the Kalman filter since it gives us the predictive densities for *x*^(*k*)^_*i*_ given inputs up to time *k* − 1.

This now enables us to solve the integral in Equation B3, yielding (see Appendix C)

(B6) $$\begin{array}{l} I\left(x_{i}^{\left(k\right)}\right)=-\frac{1}{2}\ln\left(\sigma_{i-1}^{\left(k-1\right)}+t^{\left(k\right)}\exp\left(\kappa_{i-1}x_{i}^{\left(k\right)}+\omega_{i-1}\right)\right)\\ \;-\frac{1}{2}\frac{\sigma_{i-1}^{\left(k\right)}+{\left(\mu_{i-1}^{\left(k\right)}-\mu_{i-1}^{\left(k-1\right)}\right)}^{2}}{\sigma_{i-1}^{\left(k-1\right)}+t^{\left(k\right)}\exp\left(\kappa_{i-1}x_{i}^{\left(k\right)}+\omega_{i-1}\right)}\\ \;-\frac{1}{2}\frac{1}{\sigma_{i}^{\left(k-1\right)}+t^{\left(k\right)}\exp\left(\kappa_{i}\mu_{i+1}^{\left(k-1\right)}+\omega_{i}\right)}{\left(x_{i}^{\left(k\right)}-\mu_{i}^{\left(k-1\right)}\right)}^{2}. \end{array}$$

Following the procedure of Mathys et al. (2011), we can now calculate the mean and precision of the Gaussian posterior for *x*^(*k*)^_*i*_:

(B7) $$\pi_{i}^{\left(k\right)}=-I^{\prime \prime }\left({\hat{\mu }}_{i}^{\left(k\right)}\right),$$

(B8) $$\mu_{i}^{\left(k\right)}={\hat{\mu }}_{i}^{\left(k\right)}+\frac{1}{\pi_{i}^{\left(k\right)}}I^{\prime }\left({\hat{\mu }}_{i}^{\left(k\right)}\right),$$

where a double and single prime denote second and first derivative, respectively. This gives us Equations 9 and 10.

### (C) calculation of the variational energy

By substituting Equation B5 into Equation B3, we get

We now have to solve these last two integrals. The first one can be solved analytically:

To solve the second integral, we take

The sum of Equations C2 and C3 then is the variational energy of Equation B6, up to a constant term that can be absorbed into the normalization constant  (cf. Equation B2).

### (D) variational energies for categorical outcomes

Using the notation

(D1) $${\langle f(x)\rangle }_{q_{j}}\overset{\text{def}}{=}\int q\left(x_{j}\right)f(x)\text{d}x_{j}$$

for the expectation of *f*(*x*) under *q*(*x*_*j*_) together with the definition of the model described graphically **Figure 2**, we can rewrite Equation B3 as a sum of expectations

(D2) $$\begin{array}{l} I\left(x_{i}^{\left(k\right)}\right)={\langle \ln p\left(u^{\left(k\right)},x^{\left(k\right)}|\chi \right)\rangle }_{q_{\backslash i}}\\ \;={\langle \ln p\left(u^{\left(k\right)}|x_{1}^{\left(k\right)}\right)\rangle }_{q_{\backslash i}}+{\langle \ln p\left(x_{1}^{\left(k\right)}|x_{2}^{\left(k\right)}\right)\rangle }_{q_{\backslash i}}\\ \;+{\langle \ln p\left(x_{2}^{\left(k\right)}|x_{3}^{\left(k\right)},\kappa ,\omega \right)\rangle }_{q_{\backslash i}}+{\langle \ln p\left(x_{3}^{\left(k\right)}|\vartheta \right)\rangle }_{q_{\backslash i}} \end{array}$$

The term *p* (*u*^(*k*)^ | *x*^(*k*)^_1_) is included here to cover also models with sensory uncertainty as discussed in Mathys et al. (2011). In cases without such uncertainty (sc. *x*_1_ = *u*), the term vanishes. *p* (*x*^(*k*)^_1_ | *x*^(*k*)^_2_) can be taken directly from Equation 5, while

and

This is the “prediction step” (cf. Appendix B).

We only need to determine the *I* (*x*^(*k*)^_*i*_) up to a constant because any constant term can always be absorbed into  when forming $\hat{q}$(*x*^(*k*)^_*i*_) according to Equation B2. For the three levels of our example model, this means

(D5) $$I\left(x_{1}^{\left(k\right)}\right)={\langle \ln p\left(u^{\left(k\right)}|x_{1}^{\left(k\right)}\right)\rangle }_{q_{\backslash 1}}+{\langle \ln p\left(x_{1}^{\left(k\right)}|x_{2}^{\left(k\right)}\right)\rangle }_{q_{\backslash 1}}+\text{const}.$$

(D6) $$I\left(x_{2}^{\left(k\right)}\right)={\langle \ln p\left(x_{1}^{\left(k\right)}|x_{2}^{\left(k\right)}\right)\rangle }_{q_{\backslash 2}}+{\langle \ln p\left(x_{2}^{\left(k\right)}|x_{3}^{\left(k\right)},\kappa ,\omega \right)\rangle }_{q_{\backslash 2}}+\text{const}.$$

(D7) $$I\left(x_{3}^{\left(k\right)}\right)={\langle \ln p\left(x_{2}^{\left(k\right)}|x_{3}^{\left(k\right)},\kappa ,\omega \right)\rangle }_{q_{\backslash 3}}+{\langle \ln p\left(x_{3}^{\left(k\right)}|\vartheta \right)\rangle }_{q_{\backslash 3}}+\text{const}.$$

With two exceptions, all integrals on the right-hand sides above can be solved analytically in all cases considered here, including sensory uncertainty and inference on continuous-valued states.

The two exceptions are the following: first, to solve 〈ln *p* (*x*^(*k*)^_1_ | *x*^(*k*)^_2_)〉_*q*_\1__, we expand *s* (*x*^(*k*)^_2_) to second order around the prior expectation μ^(*k* − 1)^_2_ of *x*^(*k*)^_2_:

(D8) $$\begin{array}{l} \ln s\left(x_{2}^{\left(k\right)}\right)\approx \ln s\left(\mu_{2}^{\left(k-1\right)}\right)+\left(1-s\left(\mu_{2}^{\left(k-1\right)}\right)\right)\left(x_{2}^{\left(k\right)}-\mu_{2}^{\left(k-1\right)}\right)\\ \;+\frac{1}{2}\left(s{\left(\mu_{2}^{\left(k-1\right)}\right)}^{2}-s\left(\mu_{2}^{\left(k-1\right)}\right)\right){\left(x_{2}^{\left(k\right)}-\mu_{2}^{\left(k-1\right)}\right)}^{2} \end{array}$$

Second, to solve 〈ln *p* (*x*^(*k*)^_2_ | *x*^(*k*)^_3_, κ, ω)〉_*q*\_2__, we take

(D9) $${\langle \frac{1}{\sigma_{2}^{\left(k-1\right)}+t^{\left(k\right)}\exp\left(\kappa \cdot x_{3}^{\left(k\right)}+\omega \right)}\rangle }_{q_{\backslash 2}}\approx \frac{1}{\sigma_{2}^{\left(k-1\right)}+t^{\left(k\right)}\exp\left(\kappa \cdot \mu_{3}^{\left(k-1\right)}+\omega \right)}$$

The result of doing the integrals in Equations D5–D7 is:

(D10) $$I\left(x_{1}^{\left(k\right)}\right)=x_{1}^{\left(k\right)}\ln s\left(\mu_{2}^{\left(k-1\right)}\right)+\left(1-x_{1}^{\left(k\right)}\right)\ln\left(1-s\left(\mu_{2}^{\left(k-1\right)}\right)\right)$$

(D11) $$I\left(x_{2}^{\left(k\right)}\right)=\ln s\left(x_{2}^{\left(k\right)}\right)+x_{2}^{\left(k\right)}\left(\mu_{1}^{\left(k\right)}-1\right)-\frac{1}{2\left(\sigma_{2}^{\left(k-1\right)}+{\text{e}}^{\kappa \mu_{3}^{\left(k-1\right)}+\omega }\right)}{\left(x_{2}^{\left(k\right)}-\mu_{2}^{\left(k-1\right)}\right)}^{2}$$

(D12) $$\begin{array}{l} I\left(x_{3}^{\left(k\right)}\right)=-\frac{1}{2}\ln\left(\sigma_{2}^{\left(k-1\right)}+{\text{e}}^{\kappa x_{3}^{\left(k\right)}+\omega }\right)-\frac{1}{2}\frac{\sigma_{2}^{\left(k\right)}+{\left(\mu_{2}^{\left(k\right)}-\mu_{2}^{\left(k-1\right)}\right)}^{2}}{\sigma_{2}^{\left(k-1\right)}+{\text{e}}^{\kappa x_{3}^{\left(k\right)}+\omega }}\\ \;-\frac{1}{2\left(\sigma_{3}^{\left(k-1\right)}+\vartheta \right)}{\left(x_{3}^{\left(k\right)}-\mu_{3}^{\left(k-1\right)}\right)}^{2}. \end{array}$$

At the first level, *q* can be calculated directly. By inserting Equation D10 into Equation B2 and noting that, at the first level, *q* = $\hat{q}$ since $\hat{q}$ already conforms to the distributional assumptions (Bernoulli) that *q* is subject to, we find

(D13) $$q\left(x_{1}^{\left(k\right)}\right)=\{\begin{array}{c} \begin{array}{l} 1\text{ for }x_{1}^{\left(k\right)}=u^{\left(k\right)},\\ 0\text{ otherwise}. \end{array} \end{array}$$

This result is due to the fact that, in the absence of sensory uncertainty, *I* (*x*_1_) is only defined for *x*_1_ = *u* while taking on a value of “minus infinity” otherwise (cf. Equation D5).

At the second level, applying Equations B7 and B8 to Equation D11 yields Equations 32 and 33. At the third level, we recover the standard HGF variational energy for the top level given in Equation B6, yielding the standard HGF updates of Equations 9 and 10.

### (E) derivation of ξ^*^

ξ^*^ can be unpacked in the following way:

(E1) $$\begin{array}{l} \xi^{*}=\underset{\xi }{\text{arg max}}\;p\left(\xi |y,u\right)=\underset{\xi }{\text{arg max}}\frac{p\left(\xi ,y|u\right)}{p(y)}\\ \;=\underset{\xi }{\text{arg max}}\;p\left(\xi ,y|u\right)\;=\underset{\xi }{\text{arg max}}\ln p\left(\xi ,y|u\right)\\ \;=\underset{\xi }{\text{arg max}}\ln\left(p\left(y|\xi ,u\right)p\left(\xi \right)\right)\\ \;=\underset{\xi }{\text{arg max}}\left(\ln p\left(y|\xi ,u\right)+\ln p\left(\xi \right)\right)\\ \;=\underset{\xi }{\text{arg max}}\left(\sum_{k}\ln p\left(y^{\left(k\right)}|\xi ,u\right)+\ln p\left(\xi \right)\right)\\ \;=\underset{\xi }{\text{arg max}}\left(\sum_{k}\ln p\left(y^{\left(k\right)}|\lambda^{\left(k\right)}\left(\chi ,\lambda^{\left(0\right)},u\right),\zeta \right)+\ln p\left(\xi \right)\right) \end{array}$$

### (F) coordinate choice on higher levels

This appendix deals with the consequences of choosing a particular scale and origin for *x*_*i*_ on higher levels (i.e., where *i* is greater than 1 for *x*_1_ continuous and greater than 2 for *x*_1_ discrete). These consequences are important for parameter identifiability. Whenever, there is an ambiguity between parameter values and a coordinate transformation (i.e., shifting and rescaling) of *x*_*i*_, one or several parameters will not be indentifiable. An example will show what we mean by this.

In a three-level HGF with binary *x*_1_, the state *x*_3_ at the third level of the model represents the log-volatility of *x*_2_. We now make use of the fact that any change in the initial value μ^(0)^_3_ of μ_3_ can be neutralized by corresponding changes in κ and ω. This means that by adjusting μ^(0)^_3_ appropriately, we can set κ = 1, thereby making it seemingly disappear from the model. As an example, here are parameter estimates from a behavioral study where estimation was performed with μ^(0)^_3_ set to 1:

(F1) $$\begin{array}{l} \;\kappa =2.49\\ \sigma_{3}^{\left(0\right)}=0.988\\ \;\vartheta =0.000592 \end{array}$$

We can now make κ disappear by setting it to 1. This change should be neutral to the model's predictions of input, which can only be achieved by the following compensatory substitutions:

(F2) $$\begin{array}{l} \;\mu_{3}^{\left(0\right)}=1\to \mu_{3}^{\left(0\right)}=2.49\\ \;\sigma_{3}^{\left(0\right)}=0.988\to \sigma_{3}^{\left(0\right)}=0.988\cdot 2.49^{2}\\ \vartheta =0.000592\to \vartheta =0.000592\cdot 2.49^{2} \end{array}$$

At the first two levels, nothing has changed (the trajectory of μ_2_ and therefore the input predictions $\hat{\mu }$_1_ are the same); however, at the third level *x*_3_ has been rescaled by the inverse of the factor with which κ has been rescaled (i.e., 1/2.49). This means that the term

(F3) $$v^{\left(k\right)}\overset{\text{def}}{=}t^{\left(k\right)}\exp\left(\kappa \mu_{3}^{\left(k-1\right)}+\omega \right)$$

is invariant under the above transformation for all time points *k*, leading to the same trajectory in μ_2_ and σ_2_ according to Equations 37 and 38 as before.

Effectively, because of the transformation to a fixed κ = 1, our estimate of κ has been reinterpreted as an estimate of μ^(0)^_3_. This is a consequence of our freedom to choose coordinates on *x*_3_. To take an analogy from geometry, the distance between Zurich and London is an objective geometric quantity that does not change whether it is measured in miles or kilometers; we may even introduce new units where this distance is 1. Likewise, we may always rescale *x*_3_ individually for each agent (and estimation) such that the coupling κ between the second and third level has value 1. Note, however, that this does not prevent the coupling from differing between agents, just as the actual geometric lengths of a mile and a kilometer are different even though they both have value 1 in a mile-based and kilometer-based coordinate system, respectively.

Whenever the representations of *x*_3_ (i.e., μ_3_ and σ_3_) are part of the observation model, this gives us a direct handle on *x*_3_ and measures of its representations can immediately be compared between agents, provided we use always the same coordinates (which we would automatically do without even thinking about it; individually rescaling κ to 1 would then obviously be unwise). However, in cases where we have no measure of *x*_3_ through the observation model, there is a fundamental ambiguity between individual differences in coupling (i.e., κ) and individual differences in priors for *x*_3_ (i.e., μ^(0)^_3_).

Nonetheless, we have to make some choice of coordinates. In setting μ^(0)^_3_ = 1, we choose to take the belief on environmental volatility that an agent begins inference with as the benchmark. Observed differences in learning are then attributed to, inter alia, differences in coupling. This is one way to obtain comparable measures of belief on *x*_3_ (and consequently, ?) between agents, since one may be able to influence their priors while there is usually no way to equalize their coupling levels.

Just as it is possible to set κ to an arbitrary non-zero value while keeping *v* invariant by compensatory substitutions, one can set ω to an arbitrary value with invariant *v* using another set of compensatory substitutions. In particular, ω can be set to zero, thereby making it seemingly disappear from the model, just as κ seems to disappear when set to 1.

We have seen that any change of scale in *x*_3_ is expressed in a corresponding change of κ. However, there is an additional degree of freedom in choosing coordinates on *x*_3_: the choice of origin. Changes in this are expressed in a corresponding change of ω:

(F4) $$x_{3}^{\prime }\overset{\text{def}}{=}x_{3}+a$$

then

(F5) $$\exp\left(\kappa x_{3}+\omega \right)=\exp\left(\kappa \left(x_{3}^{\prime }-a\right)+\omega \right)=\exp\left(\kappa x_{3}^{\prime }+\omega^{\prime }\right)$$

with

(F6) $$\omega^{\prime }\overset{\text{def}}{=}-\kappa a+\omega$$

In our example, we have reinterpreted the estimate of κ as one of μ^(0)^_3_. We may now go on to reinterpret it another time, this time as an estimate of ω (up to now fixed to –4) by shifting the origin on *x*_3_ such that μ^(0)^_3_ is again fixed to 1:

(F7) $$\begin{array}{l} \mu_{3}^{\left(0\right)}=2.49\to \mu_{3}^{\left(0\right)}=1\\ \;\omega =-4\to \omega =-\kappa \left(1-2.49\right)+\left(-4\right)=-2.51 \end{array}$$

Again, the trajectories at the first two levels have not changed. It is now apparent that by rescaling *x*_3_ and shifting its origin, we can choose arbitrary values for two out of the three parameters μ^(0)^_3_, κ, and ω. However, we repeat that this is only possible as long as we do not measure *x*_3_ on any objective scale by including its representations in the response model.

Equivalence classes (with equivalence defined as leading to invariant *v*) of parameter values are defined by the following *conservation laws*:

(F8) $$\begin{array}{l} \kappa^{\prime }\mu_{3}^{\prime (0)}+\omega^{\prime }=\kappa \mu_{3}^{\left(0\right)}+\omega \\ \;\kappa^{\prime 2}\vartheta^{\prime }=\kappa^{2}\vartheta \\ \;\kappa^{\prime 2}\sigma_{3}^{\prime (0)}=\kappa^{2}\sigma_{3}^{\left(0\right)} \end{array}$$

Using these equations, we can now make κ and ω seemingly disappear by setting them to 1 and 0, respectively. Note that this does not amount to a simplification of the model: it is only a coordinate choice. In our example this means:

(F9) $$\begin{array}{l} \omega =-2.51\to \omega =0\\ \;\mu_{3}^{\left(0\right)}=1\to \mu_{3}^{\left(0\right)}=-1.51 \end{array}$$

This disappearance of κ and ω (and in the general case, all κ_*i*_ and ω_*i*_) from the model may seem convenient. However, a danger here is to confuse coordinates with the underlying reality they describe. Crucially, it is impossible to discuss the choice of coordinate choice on higher levels in a model that lacks κ_*i*_ and ω_*i*_. Yet this choice does not cease to exist only because one makes it implicitly instead of explicitly.

In summary, in order to avoid on overparameterized model when μ_*i*_ is not included in the response model, it is advisable to be explicitly arbitrary by fixing two out of μ^(0)^_*i*_, κ_*i*_, and ω_*i*_, reflecting a choice of origin and scale on *x*_*i*_. By contrast, when μ_*i*_ is included in the response model, all of μ^(0)^_*i*_, κ_*i*_, and ω_*i*_ can be estimated.
